# SLX4IP Antagonizes Promiscuous BLM Activity during ALT Maintenance

**DOI:** 10.1016/j.molcel.2019.07.010

**Published:** 2019-10-03

**Authors:** Stephanie Panier, Marija Maric, Graeme Hewitt, Emily Mason-Osann, Himabindu Gali, Anqi Dai, Adam Labadorf, Jean-Hugues Guervilly, Philip Ruis, Sandra Segura-Bayona, Ondrej Belan, Paulina Marzec, Pierre-Henri L. Gaillard, Rachel L. Flynn, Simon J. Boulton

**Affiliations:** 1 The Francis Crick Institute, 1 Midland Road, London NW1 1AT, UK; 2 Centre de Recherche en Cancérologie de Marseille, CRCM, CNRS, Aix Marseille Université, INSERM, Institut Paoli-Calmettes, 27 Boulevard Leï Roure, 13009 Marseille, France; 3 Boston University School of Medicine, 72 East Concord Street, Boston, MA 02118, USA

**Keywords:** SLX4IP, SLX4, BLM, XPF, homologous recombination, ALT, telomere, genome stability, cancer

## Abstract

Cancer cells acquire unlimited proliferative capacity by either re-expressing telomerase or inducing alternative lengthening of telomeres (ALT), which relies on telomere recombination. Here, we show that ALT recombination requires coordinate regulation of the SMX and BTR complexes to ensure the appropriate balance of resolution and dissolution activities at recombining telomeres. Critical to this control is SLX4IP, which accumulates at ALT telomeres and interacts with SLX4, XPF, and BLM. Loss of SLX4IP increases ALT-related phenotypes, which is incompatible with cell growth following concomitant loss of SLX4. Inactivation of BLM is sufficient to rescue telomere aggregation and the synthetic growth defect in this context, suggesting that SLX4IP favors SMX-dependent resolution by antagonizing promiscuous BLM activity during ALT recombination. Finally, we show that SLX4IP is inactivated in a subset of ALT-positive osteosarcomas. Collectively, our findings uncover an SLX4IP-dependent regulatory mechanism critical for telomere maintenance in ALT cancer cells.

## Highlights

• SLX4IP is a regulator of ALT telomere maintenance
• SLX4IP interacts with SLX4, XPF, and BLM
• SLX4IP and SLX4 prevent BLM-dependent telomere aggregation
• SLX4IP is inactivated in a subset of ALT-positive osteosarcomas

## Graphical Abstract

**Figure:**
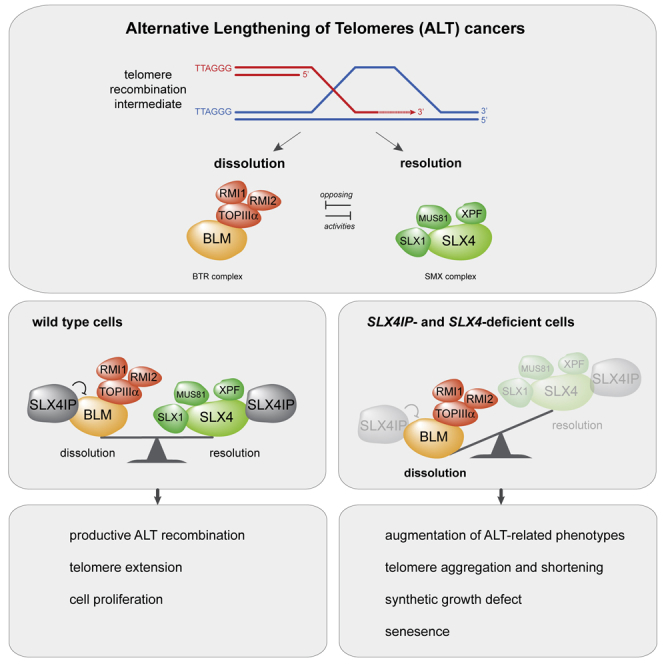

## Teaser

Panier et al. reveal that SLX4IP is a regulator of ALT telomere maintenance that binds to both BLM and SLX4 and influences the balance between resolution and dissolution at recombining telomeres. Its importance for the ALT process is underscored by the finding that SLX4IP is inactivated in a subset of ALT-positive osteosarcomas.

## Introduction

Genome stability is essential for cells to function properly and to ensure the survival of the organism. The ends of linear chromosomes are protected and maintained by nucleoprotein structures called telomeres. In vertebrates, telomeres consist of long double-stranded stretches of 5′-(TTAGGG)-3′ repeats, which end in a 3′ single-stranded DNA overhang that folds back and invades its complementary strand to form a T-loop (Allshire et al., 1988, de Lange, 2005, Makarov et al., 1997, Moyzis et al., 1988).

In somatic cells, telomeres progressively shorten after DNA replication, which ultimately results in replicative senescence and cell death (Chin et al., 1999). In contrast, tumor cells must counteract telomere attrition to achieve replicative immortality and do so by activating one of two distinct telomere maintenance mechanisms. The first mechanism is based on the re-expression of the reverse transcriptase telomerase, which synthesizes new telomeric sequence from its own RNA template (Greider and Blackburn, 1985, Greider and Blackburn, 1987). Approximately 85%–90% of tumors rely on this mechanism (Shay and Bacchetti, 1997). The second mechanism, known as alternative lengthening of telomeres (ALT), extends telomeres by upregulating homology-directed recombination pathways (Bryan et al., 1995, Bryan et al., 1997, Dunham et al., 2000, Lundblad and Blackburn, 1993, Shay and Bacchetti, 1997). ALT-positive tumors account for approximately 10%–15% of all tumors and are particularly prevalent in tumors of mesenchymal origin (Heaphy et al., 2011b, Henson and Reddel, 2010). These ALT cancers are mostly associated with a poor prognosis because of their complex karyotype and lack of targeted therapies (Dilley and Greenberg, 2015).

ALT-positive cells are characterized by several defining characteristics that include telomere recombination, heterogeneous telomere lengths, extrachromosomal telomeric DNA, and telomeric DNA damage (Bryan et al., 1995, Cesare and Griffith, 2004, Cesare et al., 2009, Londoño-Vallejo et al., 2004, Nabetani and Ishikawa, 2009). ALT telomeres also tend to cluster in a subtype of promyelocytic leukemia (PML) nuclear bodies, so-called ALT-associated PML bodies (APBs), which are potential sites of ALT-dependent telomere recombination (Draskovic et al., 2009, Yeager et al., 1999). Although the mechanisms underpinning ALT induction and maintenance are poorly understood, evidence suggests that telomeres are extended through an atypical break-induced replication (BIR) mechanism that involves strand invasion of intra- and inter-telomere sequences followed by homology-directed DNA synthesis and processing of the resulting recombination intermediates (Dilley et al., 2016).

The RecQ helicase BLM plays a central role in DNA replication and homologous recombination and as such is required for efficient telomere extension during ALT (Bhattacharyya et al., 2009, Manthei and Keck, 2013, Root et al., 2016, Stavropoulos et al., 2002). As a member of the BTR complex, which also includes TOP3α, RMI1, and RMI2, BLM catalyzes the dissolution of recombination intermediates during homologous recombination (Bussen et al., 2007, Raynard et al., 2006, Singh et al., 2008, Wu et al., 2006, Wu and Hickson, 2003, Xu et al., 2008). BLM activity is counterbalanced by the SMX complex, which promotes the resolution of recombination intermediates (Castor et al., 2013, Guervilly and Gaillard, 2018, Sarkar et al., 2015, Sobinoff et al., 2017, Wechsler et al., 2011, Wyatt et al., 2013). The SMX complex is composed of the SLX4 scaffolding protein and the structure-specific endonucleases SLX1, MUS81-EME1, and XPF-ERCC1 (Fekairi et al., 2009, Muñoz et al., 2009, Svendsen et al., 2009). SMX is recruited to telomeres through a direct interaction between SLX4 and the telomeric shelterin component TRF2 and has been implicated in telomere recombination and processing in ALT-negative cells (Muñoz et al., 2005, Saint-Léger et al., 2014, Svendsen et al., 2009, Vannier et al., 2009, Wan et al., 2013, Wilson et al., 2013, Wu et al., 2008, Zeng et al., 2009, Zhu et al., 2003). How the opposing activities of the BTR and SMX complexes are controlled in the context of ALT telomeres remains unclear.

Here we report that the uncharacterized protein SLX4IP engages with ALT telomeres and uniquely interacts with both the SMX and BTR complexes. Although SLX4IP is dispensable for telomere maintenance in telomerase-positive cells, its loss in ALT cells confers telomere hyper-recombination. This is further exacerbated by co-depletion of SLX4, leading to entangled telomeres and a synthetic growth defect. Strikingly, the detrimental effect of combined loss of SLX4 and SLX4IP in ALT cells can be rescued by removing BLM. We propose that SLX4IP counteracts promiscuous BLM activity to ensure the appropriate processing of ALT telomeres by the SMX complex. The clinical importance of SLX4IP in the ALT process is highlighted by its inactivation in a subset of ALT-positive osteosarcomas.

## Results

### SLX4IP Localizes at Telomeres in an SLX4-Dependent Manner

SLX4IP was first identified as interacting with SLX4 but has remained functionally uncharacterized (Svendsen et al., 2009). To explore a potential role for SLX4IP in the maintenance of genome stability, we first analyzed the localization of SLX4IP in the presence of DNA-damaging agents. We found that GFP-tagged SLX4IP weakly accumulates at microlaser-induced DNA damage tracks (Figure S1A). Furthermore, endogenous SLX4IP showed weak co-localization with the DNA damage marker γ-H2AX in cells treated with the DNA inter-strand crosslinking agent mitomycin C (MMC) (Figures S1B and S1C) but not in cells treated with the topoisomerase I inhibitor camptothecin (CPT) (Figures S1D and S1E).

**Figure 1:**
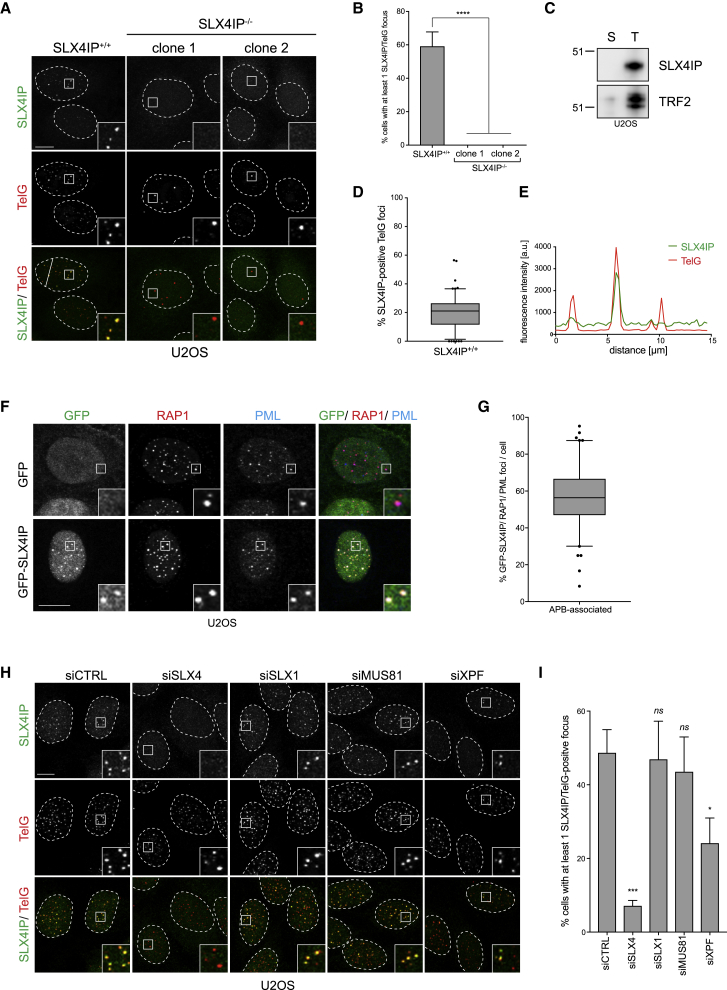
SLX4IP Localizes at Telomeres in an SLX4-Dependent Manner (A) U2OS cells were fixed and processed for SLX4IP immunofluorescence followed by telomeric PNA (TelG) FISH. Scale bar represents 10 μm. Line across the nucleus in SLX4IP^+/+^ indicates line profile measured in (D). Dashed lines indicate nucleus outlines (as determined using DAPI staining; not shown). Insets represent 3× magnifications of the indicated fields. (B) Quantification of (A). At least 100 cells per condition were counted. Data are represented as mean ± SD; n = 3; ^∗∗∗∗^p < 0.00001, Student’s t test. (C) Chromatin was isolated from whole-cell U2OS extracts with either a scrambled control (S) or a telomere-specific (T) 2′F-RNA probe. The chromatin was separated using SDS-PAGE and analyzed using SLX4IP immunoblotting. TRF2 was used as a telomeric chromatin control. Numbers denote molecular weight (kDa). (D) Quantification of (A). At least 70 cells per experiment were counted. Data are represented as mean ± SD; n = 3. (E) A random straight line was drawn across through a single Z section of the nucleus shown in SLX4IP^+/+^ in (A). The intensity of SLX4IP and TelG (telomeric PNA probe) was quantitated along the length of the line to generate a line profile. (F) U2OS cells transfected with GFP or GFP-SLX4IP were fixed and processed for GFP, RAP1, and PML immunofluorescence. Scale bar represents 10 μm. Insets represent 3× magnifications of the indicated fields. (G) Quantification of (F). At least 50 cells per condition were counted. Data are represented as mean ± SD; n = 3. (H) U2OS cells were transfected with the indicated small interfering RNAs (siRNAs), fixed and processed for SLX4IP immunofluorescence followed by telomeric PNA (TelG) FISH. Scale bar represents 10 μm. Dashed lines indicate nucleus outlines (as determined using DAPI staining; not shown). Insets represent 3× magnifications of the indicated fields. (I) Quantification of (E). At least 100 cells per condition were counted. Data are represented as mean ± SD; n = 3; ^∗^p < 0.01 and ^∗∗∗^p < 0.0001, Student’s t test. See also Figures S1–S3.

Sub-cellular localization studies in unchallenged cells revealed that SLX4IP is chromatin bound (Figure S1F) and accumulates in sub-nuclear foci in wild-type (WT) U2OS cells (Figures 1A and 1B), which were abolished in SLX4IP^−/−^ CRISPR-knockout U2OS clones (Figures 1A and 1B; Figure S1G). Intriguingly, SLX4IP foci overlapped with a peptide-nucleic acid (PNA) telomeric DNA probe and with shelterin subunit RAP1 foci, suggesting that SLX4IP associates with telomeres (Figures 1A and 1B; Figures S1H and S1I). In agreement with proteomics of isolated chromatin segments (PICh) data (Déjardin and Kingston, 2009), SLX4IP was found to be enriched on telomeric chromatin from ALT-positive U2OS and WI38VA13 cells (Figures 1C and S1J) but not from ALT-negative HeLa 1.2.11 cells (Figure S1K). Similar results were observed using immunofluorescence (Figures S1L–S1N). Notably, only a subset of telomeres (on average 20% per cell) stained positive for SLX4IP in ALT-positive U2OS cells (Figures 1A and 1D). When the signal intensity was measured along a straight line in a single Z section through the nucleus, SLX4IP peaks corresponded mostly with high-intensity telomere PNA (TelG) peaks (Figure 1E). Furthermore, 60% of GFP-SLX4IP foci overlapped with PML-positive telomeres in U2OS cells, suggesting that SLX4IP is enriched in APB bodies (Figures 1F and 1G; Draskovic et al., 2009).

To determine how SLX4IP is recruited to telomeres, we first tested whether SLX4 or any of its associated nucleases are required for SLX4IP localization at telomeres. Depletion of SLX4, but not MUS81 or SLX1, impaired SLX4IP focus formation in U2OS cells (Figures 1H and 1I; Figure S1O). Conversely, depleting SLX4IP did not measurably reduce the recruitment of SLX4 to telomeres (Figures S1P and S1Q). Depletion of XPF also reduced the number of SLX4IP/TelG-foci-positive cells but not to the same extent as SLX4-depleted cells (Figures 1H and 1I). The loss of SLX4IP localization at telomeres following SLX4 depletion was not due to decreased SLX4IP protein levels (Figure S1O). Although SLX4IP levels were reduced in SLX4- and XPF-depleted cells, this reduction was minimal and could not account for the loss of telomeric localization because 98% of SLX4IP foci in siCTRL cells localize to telomeres (Figure S1R). Interestingly, XPF levels but not SLX4, SLX1, or MUS81 levels are mildly reduced in SLX4IP^−/−^ cells, suggesting that SLX4IP affects XPF protein levels (Figure S1S). Consistent with the fact that SLX4 associates with telomeres through an interaction with TRF2 (Svendsen et al., 2009, Wan et al., 2013, Wilson et al., 2013), depletion of TRF2 also reduced SLX4IP telomere foci, indicating that SLX4IP cannot associate with telomeres lacking TRF2 (Figures S1T–S1V). SLX4IP did not, however, co-immunoprecipitate with TRF2 either in the presence or absence of SLX4 (Figure S1W). Collectively, our data suggest that SLX4IP is recruited to clustered telomeres in ALT-positive cells via interaction with SLX4, downstream of TRF2.

### SLX4IP Localization at Telomeres Is Dependent on Its N-Terminal Putative SIM Domains

SLX4IP was previously shown to directly interact with the first 669 amino acids of SLX4 (Svendsen et al., 2009). To further refine the nature of the SLX4IP-SLX4 interaction, we generated a series of GFP-tagged truncation constructs that span the first 669 amino acids in SLX4 (Figures S2A and S2B, constructs A–C) and carried out co-immunoprecipitation studies. A GFP-SLX4 fusion containing the last 268 amino acids of the SLX4 N-terminal fragment co-immunoprecipitated SLX4IP to levels comparable with the WT control (Figure S2C, construct C). Construct C contains a MUS312-MEI9 interaction-like region (MLR), which was previously shown to interact with the XPF endonuclease (Fekairi et al., 2009). Notably, the MLR domain alone is sufficient to co-immunoprecipitate SLX4IP to levels comparable with the WT construct (Figures S2A–S2C, MLR construct). These data suggest that the MLR domain of SLX4 not only mediates XPF binding to SLX4 but also confers interaction with SLX4IP.

The finding that XPF contributes to SLX4IP telomere localization (Figures 1H and 1I) prompted us to test whether SLX4IP might interact with XPF independently of SLX4. Indeed, GFP-SLX4IP comparably co-immunoprecipitated with XPF in siCTRL and siSLX4 cells, indicating that SLX4IP binds to XPF in an SLX4-independent manner (Figure S2D). Analysis of the interaction of SLX4IP with SLX4 and its associated nucleases in different cell cycle phases showed that SLX4IP interacts with XPF throughout the cell cycle, whereas its association with SLX4, SLX1, and MUS81 peaks in mitosis when the SMX tri-nuclease complex is formed (Figures S2E and S2F; Wyatt et al., 2013).

We next turned our attention to the identification of an SLX4 interaction motif in SLX4IP. Analysis of the predicted SLX4IP amino acid sequence failed to reveal any enzymatic or protein interaction domains except for three putative SUMO-interacting motifs (SIMs) in the N and C termini of the protein (Figures S3A and S3B). Using a series of FLAG-tagged SLX4IP truncation and deletion constructs, we found that the most N-terminal 120 amino acids of FLAG-SLX4IP were necessary (Figure S3C, constructs ΔB and ΔC) and sufficient (Figure S3C, construct A) to co-immunoprecipitate with GFP-SLX4. To test whether the putative SIM domains located in the SLX4IP N terminus contribute to the interaction with SLX4, we introduced point mutations into SLX4IP that are predicted to disrupt motif structure (L16K/V17K in putative SIM1 and V115K/V116K in putative SIM2). These mutations greatly reduced the interaction with MUS81 and abolished the interactions with SLX4, SLX1, and XPF (Figure S3D).

Finally, we analyzed whether the integrity of the SLX4IP N terminus is important for the telomeric localization of SLX4IP. SLX4IP mutants failed to accumulate at telomeres in undamaged cells, suggesting that the putative N-terminal SIMs are important for SLX4IP localization at telomeres (Figures S3E and S3F). From these results, we conclude that the SLX4- and XPF-dependent telomere recruitment of SLX4IP involves the N terminus of SLX4IP.

### Loss of SLX4IP in ALT-Positive Cells Increases ALT-Related Phenotypes

**Figure 2:**
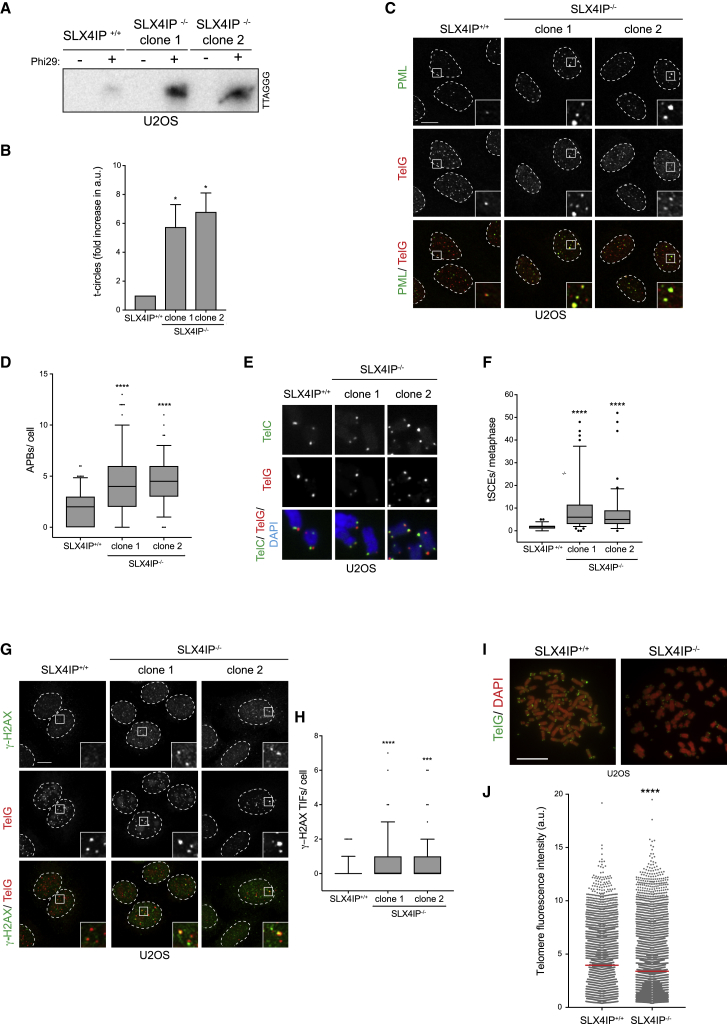
Loss of SLX4IP in ALT-Positive Cells Increases ALT-Related Phenotypes (A) Genomic DNA was isolated from U2OS cells and processed to detect Phi29-dependent telomere circles. The Phi29 amplification products were detected by Southern blotting using a γ[^32^P]-labeled telomeric (TTAGGG) probe. (B) Quantification of (A). The extent of [^32^P] incorporation was quantified from the autoradiograph and normalized to SLX4IP^+/+^, which was arbitrarily assigned a value of 1. Data are represented as mean ± SD; n = 3; ^∗^p < 0.01, Student’s t test. (C) U2OS cells were fixed and processed for PML immunofluorescence followed by telomeric PNA (TelG) FISH. Scale bar represents 10 μm. Dashed lines indicate nucleus outlines (as determined using DAPI staining; not shown). Insets represent 3× magnifications of the indicated fields. (D) Quantification of (C). At least 100 cells per condition were counted. Data are presented as 5th–95th percentiles; n = 3; ^∗∗∗∗^p < 0.00001, Student’s t test. (E) U2OS cells were fixed, and metaphases were processed for chromosome-orientation FISH using PNA probes against the C-rich (TelC) and the G-rich (TelG) telomere strand. Scale bar represents 100 μm. (F) Quantification of (E). At least 25 metaphases per condition were counted. Data are presented as 5th–95th percentiles; n = 3; ^∗∗∗∗^p < 0.00001, Student’s t test. (G) U2OS cells were fixed and processed for γ-H2AX immunofluorescence followed by telomeric PNA (TelG) FISH. Scale bar represents 10 μm. Dashed lines indicate nucleus outlines (as determined using DAPI staining; not shown). Insets represent 3× magnifications of the indicated fields. (H) Quantification of (C). At least 100 cells per condition were counted. Data are presented as 5th–95th percentiles; n = 3; ^∗∗∗^p < 0.0001 and ^∗∗∗∗^p < 0.00001, Student’s t test. (I) U2OS cells were fixed, and metaphases were processed for telomere PNA (TelG) FISH. Scale bar represents 100 μm. (J) Quantification of (H), showing the telomere fluorescence distribution of individual telomere dots. At least 25 metaphases per condition were counted. Mean fluorescence is indicated by the red horizontal line; shown is a representative experiment; ^∗∗∗∗^p < 0.00001, Student’s t test. See also Figure S4.

Prompted by the telomeric localization of SLX4IP in ALT-positive cells, we sought to analyze the consequence of deleting SLX4IP on ALT-related phenotypes, including the presence of extrachromosomal telomeric DNA circles, APBs, and telomeric sister chromatid exchanges (tSCEs). CRISPR knockouts of SLX4IP in ALT-positive U2OS and WI38VA13 cells (Figures S1G and S4C) resulted in a 6- to 8-fold increase in extrachromosomal telomere (t-) and C-circles (Figures 2A and 2B; Figures S4A–S4F), which was not seen in ALT-negative SLX4IP^−/−^ cells (Figures S4G–S4I). SLX4IP^−/−^ U2OS and WI38VA13 ALT-positive cells but not HeLa 1.2.11 ALT-negative cells exhibited an increase in the number of APB bodies per cell (Figures 2C and 2D; Figures S4J–S4M). We also analyzed the effect of SLX4IP deficiency on the frequency of tSCEs, which although not unique to ALT are common at ALT telomeres. Similar to the increase in extrachromosomal DNA circles and APB numbers, we also observed an increase in the frequency of tSCEs as assessed by chromosome-orientation FISH in SLX4IP^−/−^ U2OS cells (Figures 2E and 2F).

Additional features of ALT-positive cells include the presence of telomeric DNA damage and telomere heterogeneity. Consistent with our previous observations, SLX4IP^−/−^ U2OS cells exhibited a 2-fold increase in γ-H2AX-positive telomeres compared with SLX4IP^+/+^ cells (Figures 2G and 2H). Quantitative fluorescence *in situ* hybridization analysis (Q-FISH) of SLX4IP^−/−^ U2OS chromosome spreads also revealed that long-term loss of SLX4IP conferred enhanced telomere heterogeneity and a reduction in mean telomere length relative to SLX4IP^+/+^ U2OS cells (Figures 2I and 2J), suggesting that despite the increase in ALT-related phenotypes, telomere length is not fully maintained in SLX4IP-deficient cells.

Together, these data reveal that loss of SLX4IP in ALT-positive cell results in upregulation of ALT-related markers, whereas its removal in ALT-negative cells has no detectable impact on telomeres.

### SLX4 Depletion Further Augments the Increase in ALT-Related Phenotypes in SLX4IP^−/−^ Cells

**Figure 3:**
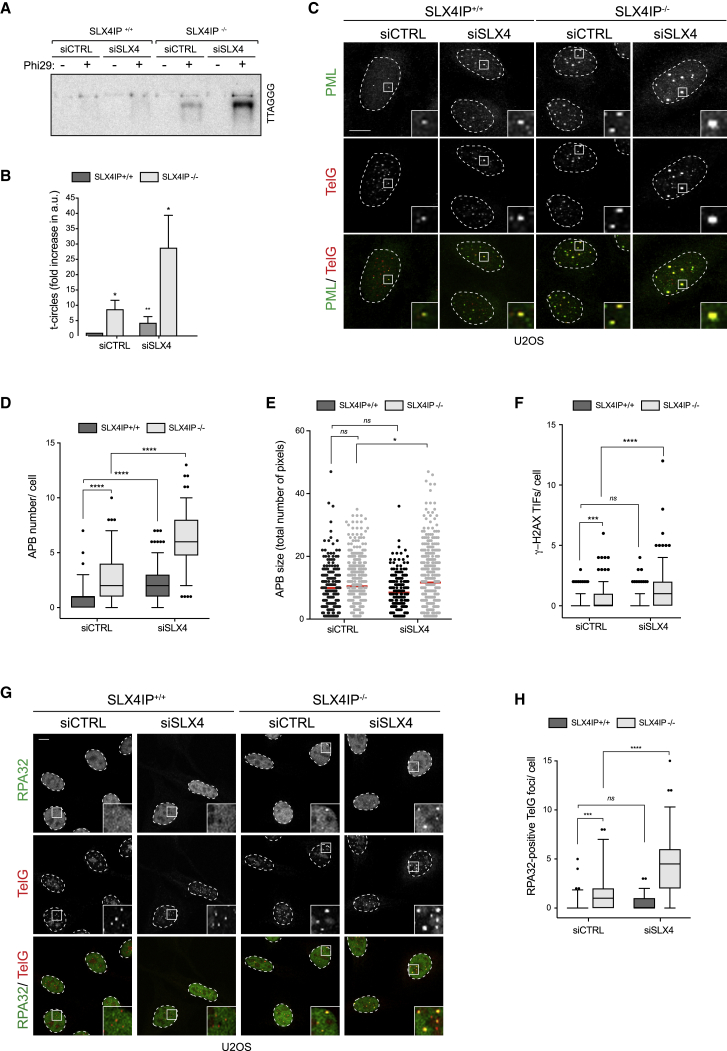
SLX4 Depletion Further Augments the Increase in ALT-Related Phenotypes in SLX4IP^−/−^ Cells (A) U2OS cells were transfected with the indicated siRNAs. Their genomic DNA was then processed to detect Phi29-dependent telomere circles. The Phi29 amplification products were detected by Southern blotting using a γ[^32^P]-labeled telomeric (TTAGGG) probe. (B) Quantification of (A). The extent of [^32^P] incorporation was quantified from the autoradiograph and normalized to SLX4IP^+/+^ siCTRL, which was arbitrarily assigned a value of 1. Data are represented as mean ± SD; n = 3; ^∗^p < 0.01, Student’s t test; ns, not significant. (C) U2OS cells transfected with the indicated siRNAs were fixed and processed for PML immunofluorescence followed by telomeric PNA (TelG) FISH. Scale bar represents 10 μm. Dashed lines indicate nucleus outlines (as determined using DAPI staining; not shown). Insets represent 3× magnifications of the indicated fields. (D) Quantification of (C). At least 100 cells per condition were counted. Data are presented as 5th–95th percentiles; n = 3; ^∗∗∗∗^p < 0.00001, one-way ANOVA. (E) Quantification of (C). APBs from at least 70 cells per condition were analyzed. Data are represented as mean ± SD; n = 2; ^∗^p < 0.01, one-way ANOVA; ns, not significant. (F) U2OS cells transfected with the indicated siRNAs were fixed and processed for γ-H2AX immunofluorescence followed by telomeric PNA FISH. At least 100 cells per condition were counted. Data are presented as 5th–95th percentiles; n = 3; ^∗∗∗^p < 0.0001 and ^∗∗∗∗^p < 0.00001, one-way ANOVA; ns, not significant. (G) U2OS cells transfected with the indicated siRNAs were fixed and processed for RPA32 immunofluorescence followed by telomeric PNA (TelG) FISH. Scale bar represents 10 μm. Dashed lines indicate nucleus outlines (as determined using DAPI staining; not shown). Insets represent 3× magnifications of the indicated fields. (H) Quantification of (G). At least 100 cells per condition were counted. Data are presented as 5th–95th percentiles; n = 3; ^∗∗∗^p < 0.0001 and ^∗∗∗∗^p < 0.00001, one-way ANOVA; ns, not significant. See also Figure S5.

Because SLX4 and SLX4IP directly interact and loss of either protein leads to an enhanced telomere phenotype in ALT-positive cells, we hypothesized that their roles at ALT telomeres would be epistatic. Contrary to expectation, we found that SLX4 depletion in SLX4IP^−/−^ U2OS cells further augmented t-circle and C-circle levels (Figures 3A and 3B; Figures S5A–S5C) and APB numbers and size (Figures 3C–3E), relative to either SLX4 or SLX4IP deficiency alone. Importantly, re-introduction of WT SLX4IP restored APB numbers back to WT levels (Figures S5D–S5F). Co-depletion of the SLX4-associated endonucleases SLX1, MUS81, and XPF did not phenocopy SLX4 depletion with regard to t-circle levels and APB numbers (Figures S5G–S5K), suggesting that the SLX4-associated endonucleases act redundantly in this context. Importantly, SLX4 depletion in SLX4IP^−/−^ ALT-negative cells did not increase t-circle levels (Figures S5L and S5M).

Further analysis of ALT-positive cells lacking both SLX4 and SLX4IP also revealed significantly enhanced numbers of γ-H2AX-positive and RPA32-positive telomeres relative to either SLX4IP or SLX4 deficiency alone (Figures 3F–3H; Figure S5N). To determine if this increase is associated with heightened telomere-associated DNA synthesis, we measured 5-ethynyl-2′-deoxyuridine (EdU) incorporation at telomeres (Dilley et al., 2016). As shown in Figures S5O and S5P, 45% of cells lacking both SLX4IP and SLX4 contained EdU-positive telomeres compared with 10% of cells lacking SLX4IP alone and 5% of WT cells. Collectively, these data indicate that loss of SLX4 further augments the ALT-related phenotypes of SLX4IP^−/−^ cells and exacerbates both recombination between telomeric sequences and telomeric DNA synthesis.

### Loss of SLX4IP and SLX4 Causes a Synthetic Growth Defect

**Figure 4:**
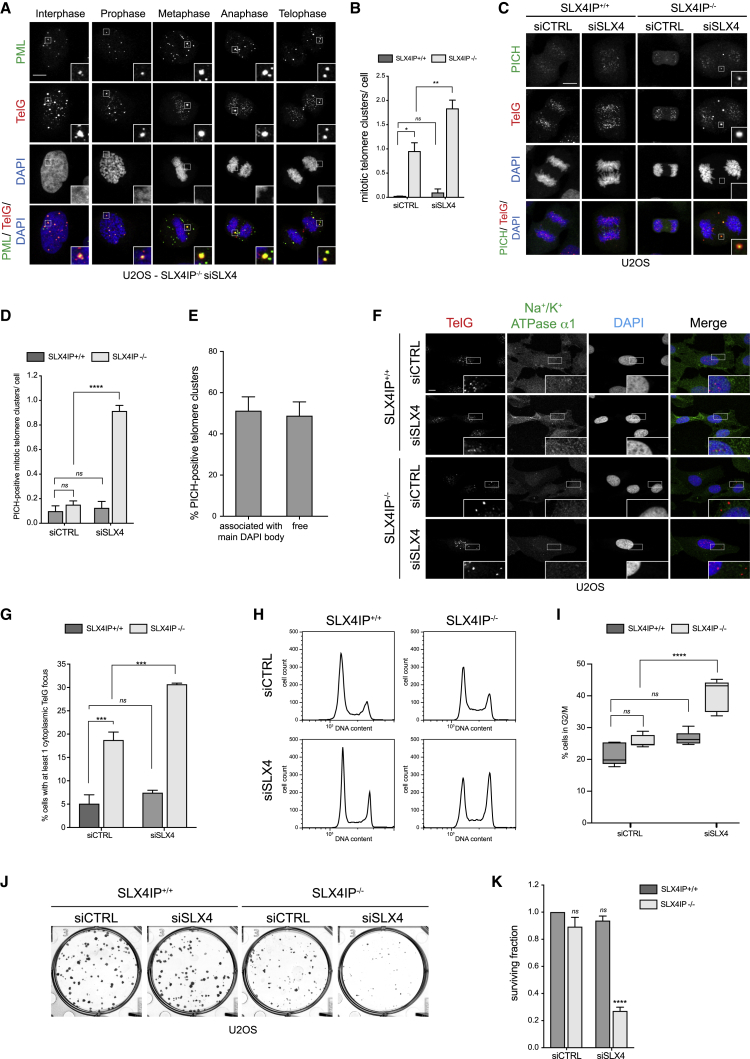
Loss of SLX4IP and SLX4 Causes a Synthetic Growth Defect (A) SLX4IP^−/−^ U2OS cells transfected with siSLX4 were fixed and processed for PML immunofluorescence followed by telomeric PNA (TelG) FISH. DNA was counterstained with DAPI. Scale bar represents 10 μm. Insets represent 3× magnifications of the indicated fields. (B) Quantification of (A). At least 30 mitotic cells per condition were counted. Data are represented as mean ± SD; n = 3; ^∗^p < 0.01 and ^∗∗^p < 0.001, one-way ANOVA; ns, not significant. (C) U2OS cells transfected with the indicated siRNAs were fixed and processed for PICH immunofluorescence followed by telomeric PNA (TelG) FISH. DNA was counterstained with DAPI. Scale bar represents 10 μm. Insets represent 3× magnifications of the indicated fields. (D) Quantification of (C). At least 30 mitotic cells per condition were counted. Data are represented as mean ± SD; n = 3; ^∗∗∗∗^p < 0.00001, one-way ANOVA; ns, not significant. (E) Quantification of (C). At least 30 mitotic cells per condition were counted. Data are represented as mean ± SD (n = 3). (F) U2OS cells transfected with the indicated siRNAs were fixed and processed for Na^+/^ K^+^ ATPase α1 immunofluorescence followed by telomeric PNA (TelG) FISH. DNA was counterstained with DAPI. Scale bar represents 10 μm. Insets represent 3× magnifications of the indicated fields. (G) Quantification of (F). At least 100 cells per condition were counted. Data are presented as mean ± SD; n = 3; ^∗∗∗^p < 0.0001, one-way ANOVA; ns, not significant. (H) U2OS cells transfected with the indicated siRNAs were fixed, stained with propidium iodide, and analyzed using FACS. At least 10,000 cells per condition were counted. (I) Quantitation of (H). Data are presented as 5th–95th percentiles; n = 5; ^∗∗∗∗^p < 0.00001, one-way ANOVA; ns, not significant. (J) U2OS cells were transfected with the indicated siRNAs. After 72 h of knockdown, cells were re-seeded and were then permitted to grow for 11 days before fixation and staining. (K) Quantitation of (I). The surviving fraction was normalized to SLX4IP^+/+^ siCTRL, which was arbitrarily assigned a value of 1. Data are represented as mean ± SD; n = 5; ^∗∗∗∗^p < 0.00001, Student’s t test; ns, not significant. See also Figure S6.

Analysis of APB-associated telomere clusters revealed a subset that persisted throughout mitosis in cells lacking both SLX4IP and SLX4 (Figures 4A and 4B). SLX4IP^−/−^ siSLX4 mitotic cells contained an average of 1.7 telomere clusters, which is a 1.7-fold increase relative to SLX4IP^−/−^ mitotic cells and a 17-fold increase relative to WT mitotic cells. Interestingly, we found that that only 45% of telomere clusters in SLX4IP^−/−^ siSLX4 cells were RPA32 positive compared with 80% in WT cells (Figures S6A and S6B). These data indicate that the telomeric clusters in SLX4IP^−/−^ siSLX4 cells not only contain extrachromosomal single-stranded telomeric DNA but are also enriched for other DNA structures. Because APBs are important for inter-telomere synapsis and ALT recombination (Cho et al., 2014), we hypothesized that the mitotic telomere clusters in SLX4IP^−/−^ siSLX4 cells might represent stalled recombination intermediates that could not be processed prior to mitosis. We reasoned that these intermediates would likely include catenated structures and therefore tested for the presence of the ATP-dependent translocase PICH, which binds to catenated DNA during mitosis (Baumann et al., 2007, Biebricher et al., 2013). This experiment revealed that SLX4IP^−/−^ siSLX4 mitotic cells contained on average 0.9 PICH-positive telomere clusters, while SLX4IP^+/+^ cells or cells lacking either SLX4IP or SLX4 contained only up to 0.2 PICH-positive telomere clusters (Figures 4C and 4D).

Because approximately 50% of PICH-positive mitotic telomere clusters were not associated with the main DAPI body (Figure 4E), we sought to understand the fate of these telomere clusters when cells re-enter interphase following mitosis. We first quantified the occurrence of telomeric DNA in the cytoplasm by labeling the plasma membrane with an antibody against alpha-1 sodium/potassium ATPase and staining the nucleus with DAPI. We found that 20% of SLX4IP^−/−^ cells and 30% of SLX4IP^−/−^ siSLX4 cells contained at least one cytoplasmic telomere focus compared with 5% of SLX4IP^+/+^ cells (Figures 4F and 4G). We also noticed that the mitotic index in SLX4IP^−/−^ siSLX4 cells was reduced by more than 50% relative to WT cells (Figure S6C). In addition, immunoblotting of whole-cell extracts of U2OS cells revealed significantly lower levels of the mitotic marker pH3 (Ser10) in SLX4IP^−/−^ siSLX4 cells (Figure S6D). Consistent with these findings, cell cycle analysis by fluorescence-activated cell sorting (FACS) revealed a G2/M arrest in SLX4IP^−/−^ siSLX4 cells, which contrasted with the normal cell cycle progression in WT U2OS cells or in cells lacking either SLX4IP or SLX4 alone (Figures 4H and 4I).

Prompted by the robust G2/M arrest, we next tested whether depleting SLX4 in SLX4IP^−/−^ cells affected clonogenic survival. As shown in Figures 4J and 4K, lack of SLX4IP or SLX4 alone did not significantly affect clonogenic survival relative to WT U2OS cells. In contrast, the combined loss of SLX4IP and SLX4 reduced the surviving fraction by 70%. Importantly, re-introduction of WT SLX4IP rescued the cell growth defect of SLX4IP^−/−^ siSLX4 cells to near WT levels (Figures S6E, S6F, and S5F). Depletion of any of the SMX nucleases in the context of SLX4IP deficiency did not affect clonogenic survival of U2OS cells (Figures S6G, S6H, S5A, and S5G). Moreover, co-depletion of SLX4 in SLX4IP^−/−^ HeLa 1.2.11 cells did not affect clonogenic survival, further supporting the idea that the telomere phenotypes we observe following SLX4IP inactivation are ALT specific (Figures S6I, S6J, and S5L).

To determine if the reduced clonogenic survival is due to the induction of apoptosis in SLX4IP^−/−^ siSLX4 cells, we immunostained with an antibody against the mitochondrial protein cytochrome *c*, which is released into the cytosol during apoptosis (Liu et al., 1996). As shown in Figure S6K, cytochrome *c* was not released in SLX4IP^−/−^ siSLX4 cells, suggesting that apoptosis is not induced in these cells. However, the cytochrome *c*-labeled mitochondria displayed an increased propensity for elongation or fusion in SLX4IP^−/−^ sSLX4 cells, which is an indicator of cellular stress and is often observed in senescent cells (Figure S6K; Mai et al., 2010, Navratil et al., 2008, Yoon et al., 2006, Zottini et al., 2006). Indeed, SLX4IP^−/−^ siSLX4 cells exhibited an 8-fold increase in the senescence marker beta-galactosidase relative to WT cells or cells lacking either SLX4IP or SLX4 alone (Figures S6L and S6M). Immunoblotting of U2OS whole-cell extracts also revealed that p62/SQTSM1, a marker of autophagic flux whose mis-regulation is linked to senescence, is increased in cells lacking either SLX4 or SLX4IP, and this increase is augmented in cells lacking both proteins (Figure S6N; Komatsu et al., 2007, Komatsu et al., 2010, Fujii et al., 2012, García-Prat et al., 2016). Hence, ALT-positive cells lacking both SLX4IP and SLX4 exhibit impaired growth and senescence.

### SLX4IP Interacts with BLM Helicase

**Figure 5:**
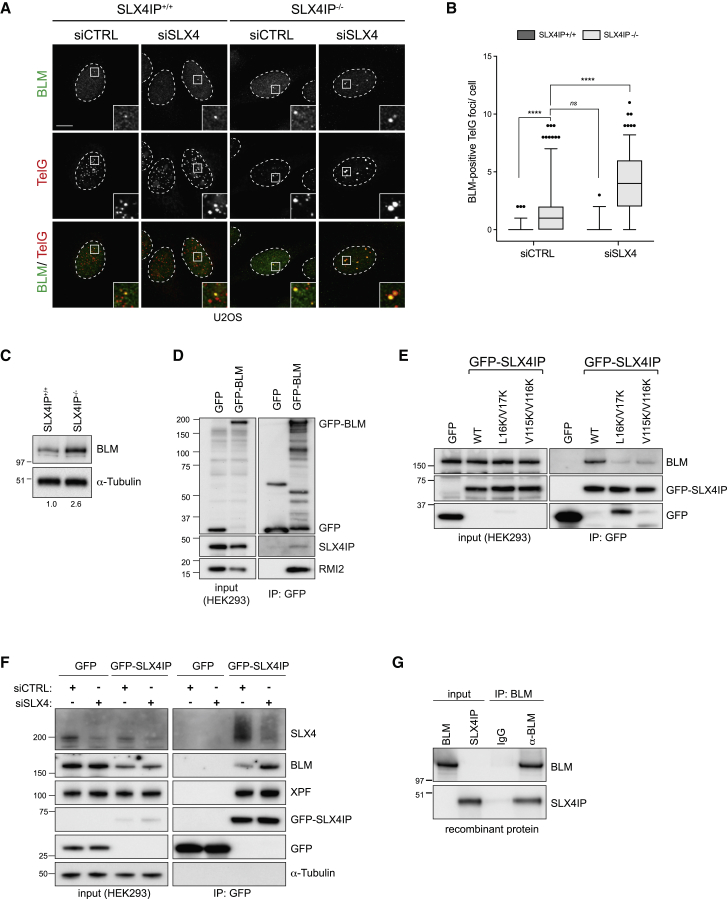
SLX4IP Interacts with BLM Helicase (A) U2OS cells transfected with the indicated siRNAs were fixed and processed for BLM immunofluorescence followed by telomeric PNA (TelG) FISH. Scale bar represents 10 μm. Dashed lines indicate nucleus outlines (as determined using DAPI staining; not shown). Insets represent 3× magnifications of the indicated fields. (B) Quantification of (A). At least 100 cells per condition were counted. Data are presented as 5th–95th percentiles; n = 3; ^∗∗∗∗^p < 0.00001, one-way ANOVA; ns, not significant. (C) U2OS whole-cell extracts were separated using SDS-PAGE and analyzed using BLM immunoblotting. Tubulin was used as loading control. Numbers on the right denote molecular weight (kDa). Numbers below indicate protein levels. Protein levels were normalized to SLX4IP^+/+^, which was arbitrarily assigned a value of 1. (D) Whole-cell extracts from HEK293 cells transiently expressing GFP constructs were subjected to GFP-trap co-immunoprecipitation (IP). Input and IP samples were separated using SDS-PAGE and analyzed using GFP, SLX4IP, and RMI2 immunoblotting. Numbers denote molecular weight (kDa). (E) Whole-cell extracts from HEK293 cells transiently expressing GFP constructs were subjected to GFP-trap co-immunoprecipitation (IP). Input and IP samples were separated using SDS-PAGE and analyzed using GFP and BLM immunoblotting. Numbers denote molecular weight (kDa). (F) Whole-cell extracts from HEK293 cells transfected with the indicated siRNAs and transiently expressing GFP constructs were subjected to GFP-trap co-immunoprecipitation (IP). Input and IP samples were separated using SDS-PAGE and analyzed using GFP, SLX4, BLM, and XPF immunoblotting. Tubulin was used as loading control. Numbers denote molecular weight (kDa). (G) Recombinant Flag-BLM and SLX4IP proteins were subjected to BLM co-immunoprecipitation (IP). Normal IgGs were used as negative IP control. Input and IP samples were separated using SDS-PAGE and analyzed using BLM and SLX4IP immunoblotting. Numbers denote molecular weight (kDa). See also Figure S7.

Our observation that SLX4IP and SLX4 are non-epistatic in ALT cells raised the possibility that SLX4IP performs SLX4-independent functions. Interestingly, in *S. pombe*, SUMOylated Rqh1, a RecQ homolog, promotes telomere breakage and entanglements in cells with dysfunctional telomeres (Rog et al., 2009). This phenotype is reminiscent of the telomere clusters observed in SLX4IP^−/−^ and SLX4IP^−/−^ siSLX4 cells and prompted us to test whether SLX4IP is functionally linked to the RecQ helicase BLM. Immunostaining showed a strong enrichment of BLM helicase at clustered SLX4IP^−/−^ siSLX4 telomeres (Figures 5A and 5B), and immunoblotting of U2OS whole-cell extracts revealed that BLM levels are elevated ∼2.5 fold in SLX4IP^−/−^ cells (Figure 5C). This increase in BLM levels was not due to changes in protein stability, because inhibition of translation with cycloheximide reduced BLM protein levels in SLX4IP^−/−^ cells at a similar rate to that observed in SLX4IP^+/+^ cells (Figures S7A and S7B). BLM mRNA levels were increased ∼2-fold in SLX4IP^−/−^ cells relative to SLX4IP^+/+^ cells, suggesting that SLX4IP-deficient cells increase the rate of *BLM* gene transcription (Figure S7C).

Co-immunoprecipitation experiments in HEK293 cells also revealed an association between endogenous SLX4IP and GFP-BLM (Figure 5D) and GFP-tagged SLX4IP and endogenous BLM (Figure 5E). This interaction was strongly reduced with the two SLX4IP SIM mutant proteins (L16K/V17K and V115K/V116K) and was also found to be resistant to Benzonase treatment, suggesting that SLX4IP and BLM are not bridged by nucleic acids (Figure S7D). Because SLX4IP binds directly to SLX4, we next asked whether the interactions of SLX4IP with BLM (and also XPF) are bridged by SLX4 (Svendsen et al., 2009). We found that both BLM and XPF co-immunoprecipitated with GFP-SLX4IP in siCTRL and siSLX4 HEK293 and U2OS cells, indicating that the interactions are independent of SLX4 (Figure 5F; Figure S7E). Finally, pull-down assays using recombinant proteins revealed that SLX4IP and BLM interact directly *in vitro* (Figure 5G; Figure S7F). Together, our data reveal that SLX4IP is physically linked to recombination resolution via SLX4 and XPF and to recombination dissolution via BLM.

### Loss of BLM Rescues the Increase in ALT-Related Phenotypes

**Figure 6:**
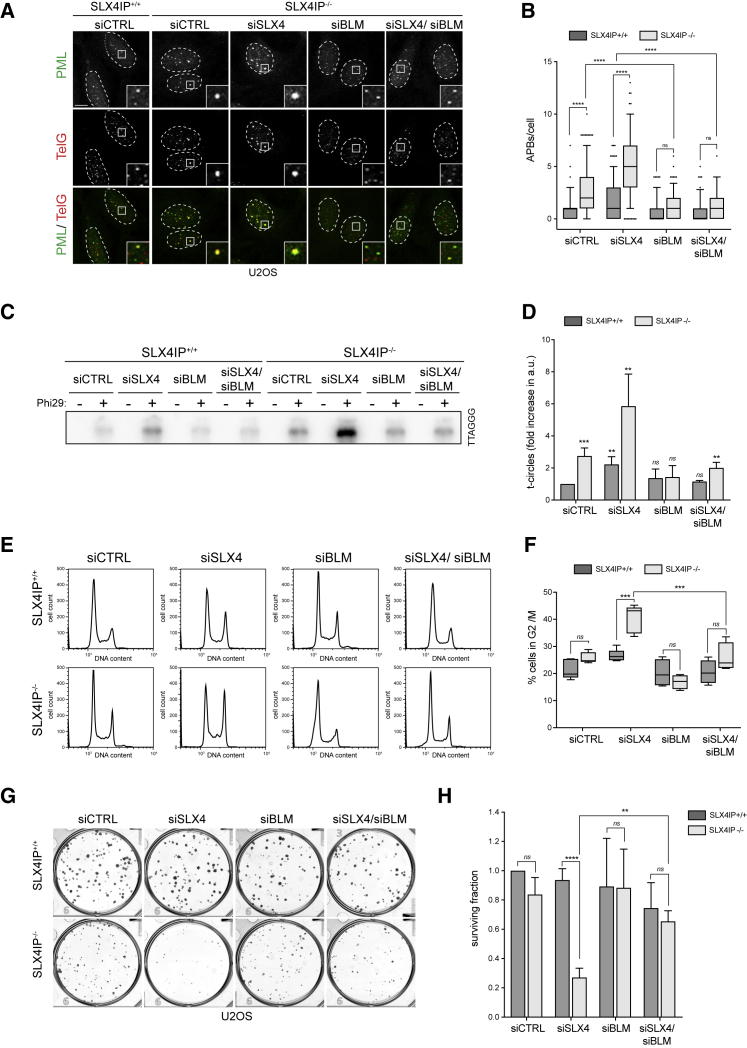
Loss of BLM Rescues the Increase in ALT-Related Phenotypes (A) U2OS cells transfected with the indicated siRNAs were fixed and processed for PML immunofluorescence followed by telomeric PNA (TelG) FISH. Scale bar represents 10 μm. Dashed lines indicate nucleus outlines (as determined using DAPI staining; not shown). Insets represent 3× magnifications of the indicated fields. (B) Quantification of (A). At least 100 cells per condition were counted. Data are presented as 5th–95th percentiles; n = 3; ^∗∗∗∗^p < 0.00001, one-way ANOVA; ns, not significant. (C) Genomic DNA was isolated from U2OS cells and processed to detect Phi29-dependent telomere circles. The Phi29 amplification products were detected by Southern blotting using a γ[^32^P]-labeled telomeric (TTAGGG) probe. (D) Quantification of (C). The extent of [^32^P] incorporation was quantified from the autoradiograph and normalized to SLX4IP^+/+^ siCTRL, which was arbitrarily assigned a value of 1. Data are represented as mean ± SD; n = 3; ^∗∗^p < 0.001 and ^∗∗∗∗^p < 0.00001, Student’s t test; ns, not significant. (E) U2OS cells transfected with the indicated siRNAs were fixed, stained with propidium iodide, and analyzed using FACS. At least 10,000 cells per condition were counted. (F) Quantification of (E). Data are presented as 5th–95th percentiles; n = 3; ^∗∗∗^p < 0.0001, one-way ANOVA; ns, not significant. (G) U2OS cells were transfected with the indicated siRNAs. After 72 h of knockdown, cells were re-seeded and were then permitted to grow for 11 days before fixation and staining. (H) Quantification of (G). The surviving fraction was normalized to SLX4IP^+/+^ siCTRL, which was arbitrarily assigned a value of 1. Data are represented as mean ± SD; n = 3; ^∗∗∗∗^p < 0.00001, Student’s t test; ns, not significant. See also Figure S7.

Because SLX4IP interacts with and affects BLM levels and concomitant loss of SLX4 augments ALT-related phenotypes in SLX4IP^−/−^ cells, we considered the possibility that the increase in ALT-related phenotypes might be caused by BLM. Strikingly, co-depletion of BLM fully rescued elevated APB numbers and t-circle levels in SLX4IP^−/−^ siCTRL and SLX4IP^−/−^ siSLX4 cells (Figures 6A–6D; Figure S7G).

Because BLM is required for both DSB end resection and recombination dissolution (Manthei and Keck, 2013), we tested whether the increase in ALT-related phenotypes of SLX4IP^−/−^ cells is dependent on exonuclease DNA2, which cooperates with BLM during DSB end resection. Depletion of DNA2 failed to rescue SLX4IP^−/−^ phenotypes and instead increased APB numbers (Figures S7H–S7J), suggesting that the telomeric phenotypes observed in SLX4IP^−/−^ cells are dependent on BLM-dependent dissolution but not on its resection activity.

We next tested whether loss of BLM could avert the cell-cycle arrest and the synthetic growth defect of SLX4IP^−/−^ siSLX4 cells. As shown in Figures 6E and 6F, the co-depletion of BLM in SLX4IP^−/−^ siSLX4 cells averted the G2/M cell-cycle arrest and resulted in a cell cycle profile that closely mirrored the profile of SLX4IP^+/+^ siCTRL cells. Co-depletion of BLM also increased the clonogenic survival of SLX4IP^−/−^ siSLX4 from 30% to 70% relative to siCTRL cells (Figures 6G and 6H), indicating that removing BLM suppresses the synthetic growth defect of SLX4IP- and SLX4-deficient cells.

### SLX4IP Is Lost in a Subset of ALT-Positive Osteosarcomas

**Figure 7:**
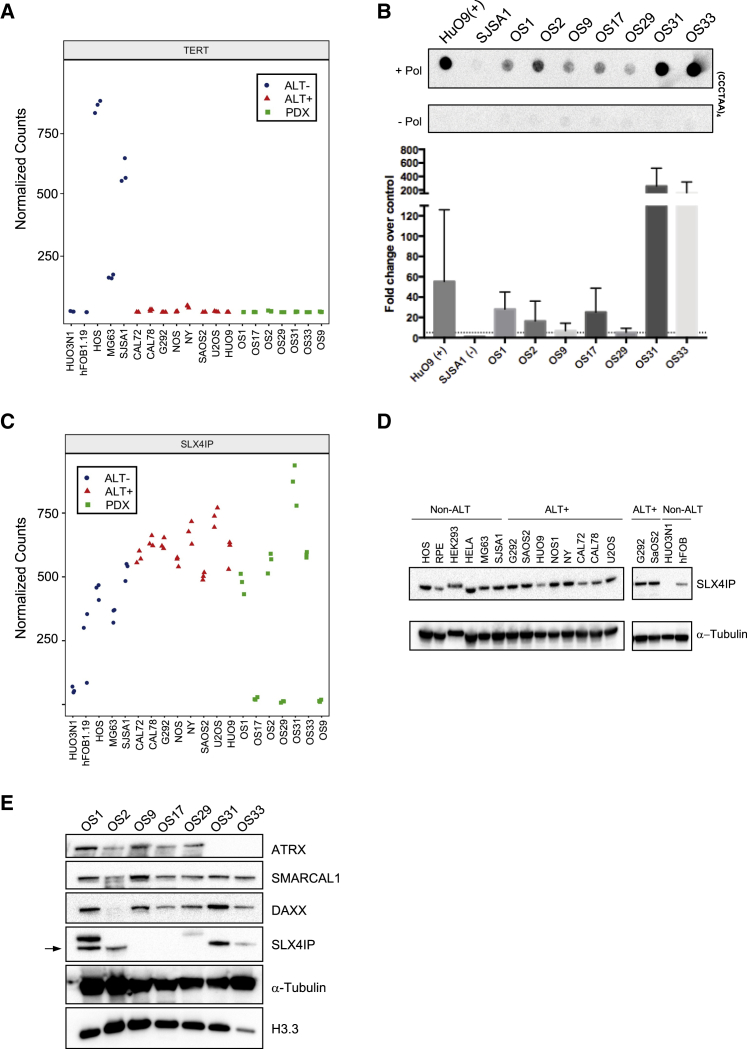
SLX4IP Is Lost in a Subset of ALT-Positive Osteosarcomas (A) Relative expression of hTERT from RNA sequencing preformed on a panel of osteosarcoma cell lines and patient-derived osteosarcoma xenografts. RNA sequencing was performed in triplicate, and each dot represents a separate experiment. (B) Quantification of C-circle abundance in the osteosarcoma PDX samples. DNA was extracted from three separate tissue sections taken from each tumor. DNA extracted from HUO9 cells was used as a positive control, and DNA extracted from SJSA1 xenografts was used as a negative control. Data are represented as mean ± SD; n = 3. Dotted line represents 5-fold change in C-circle abundance. (C) Relative expression of SLX4IP from RNA sequencing preformed on a panel of osteosarcoma cell lines and patient-derived osteosarcoma xenografts. RNA sequencing was performed in triplicate, and each dot represents a separate experiment. (D) Whole-cell extracts of the indicated cell lines were analysed by SLX4IP immunoblotting. α-Tubulin was used as loading control. (E) PDX tumor samples were analysed by SLX4IP, ATRX, SMARCAL1, DAXX and H3.3 immunoblotting. α-Tubulin was used as loading control. Arrow indicates SLX4IP band. See also Figure S7.

To date ATRX, DAXX, and SMARCAL1 are the only genes identified that regulate ALT telomere maintenance and are also found to be mutated in ALT-positive cancers (Diplas et al., 2018, Heaphy et al., 2011a, Heaphy et al., 2011b, Mason-Osann et al., 2018). In light of our findings linking SLX4IP to ALT telomere maintenance, we asked whether SLX4IP is inactivated in osteosarcoma tumors, which frequently use the ALT pathway. To this end, we analyzed the ALT status of seven osteosarcoma tumors and 13 osteosarcoma cell lines by measuring the loss of hTERT and hTERC expression and induction of C-circles. None of the seven osteosarcoma tumors demonstrated either hTERT or hTERC expression, suggesting that this subset of osteosarcoma tumors lack telomerase activity (Figure 7A; Figure S7K). Furthermore, all seven tumors exhibited abundant C-circle levels compared with xenografted telomerase-positive SJSA1 control tumors, confirming that all seven osteosarcoma tumors possess ALT activity (Figure 7B).

We then asked whether any of these tumors possess deficiencies in genes associated with ALT activity, including ATRX, DAXX, H3F3A, and SMARCAL1. Of the seven tumors, only two (OS31 and OS33) had structural variations in ATRX by RNA sequencing, while the five remaining tumors (OS1, OS2, OS9, OS17, and OS29) retained RNA expression of DAXX, SMARCAL1, and the histone variant H3F3A (H3.3) (Figure S7K). Although the *H3.3* gene is not frequently mutated in ALT-positive tumors, defects in ATRX and DAXX are believed to lead to defects in H3.3 incorporation at heterochromatic regions including telomeric DNA. Strikingly, we found that three tumors, OS9, OS17, and OS29, demonstrated loss of SLX4IP mRNA expression (Figure 7C). To confirm this result, we analyzed the seven osteosarcoma tumor samples for ATRX, DAXX, SMARCAL1, H3.3, and SLX4IP protein expression by immunoblotting (Figures 7D and 7E). As predicted from the RNA sequencing analysis, OS31 and OS33 exhibited loss of ATRX protein expression and retention of DAXX, SMARCAL1, and H3.3. Conversely, OS9, OS17, and OS29 demonstrated loss of SLX4IP protein expression while retaining ATRX, DAXX, SMARCAL1, and H3.3 protein expression. In addition to the seven tumors, we also analyzed all 13 cell lines for ATRX, DAXX, SMARCAL1, H3.3, and SLX4IP protein expression by immunoblotting (Mason-Osann et al., 2018) (Figures 7D and 7E). Consistent with the analysis in our tumor samples, SLX4IP deficiencies in our cell lines are mutually exclusive with ATRX, DAXX, SMARCAL1, and H3.3, raising the possibility that SLX4IP may represent another gene deficiency associated with ALT activity. Notably, we identified one cell line, HUO3N1, that does not maintain telomerase nor ALT activity yet is deficient for SLX4IP (Figure 7D). Collectively our data suggest that loss of SLX4IP likely contributes to the maintenance of ALT activity, but similar to ATRX, DAXX, and SMARCAL1, its loss is not sufficient to induce ALT.

## Discussion

Productive ALT recombination requires an exquisite balance between pro- and anti-recombinogenic activities. Tipping the balance in either direction causes telomere instability and impaired cell growth (Dilley and Greenberg, 2015). In the context of ALT, BLM affects telomeric DNA synthesis and processing of recombination intermediates (Bhattacharyya et al., 2009, O’Sullivan et al., 2014, Sobinoff et al., 2017, Stavropoulos et al., 2002). Despite its importance for productive ALT, BLM activity needs to be counterbalanced by SMX-mediated recombination intermediate resolution to prevent telomere breakage and entanglements. Our results implicate SLX4IP in the ALT process, in which it plays a pivotal role in opposing pathological DNA processing by BLM.

Prior to this study, SLX4IP was known to interact with SLX4, but its function had not been investigated. We show here that SLX4IP engages with telomeres primarily in ALT cells. Its recruitment to ALT telomeres is dependent on TRF2, SLX4, and XPF. Consistent with the localization of SLX4IP to ALT telomeres, loss of SLX4IP in non-ALT cells did not induce ALT or any measurable telomere phenotype. However, similar to loss of SLX4, deletion of SLX4IP in ALT-positive cell lines enhanced ALT-related phenotypes. We also observed an increase in telomere sister chromatid exchanges in SLX4IP^−/−^ cells that, although common at ALT telomeres, can be negatively correlated with productive ALT telomere extension (Sobinoff et al., 2017).

Contrary to our expectation, depletion of SLX4 further augmented the increased ALT-related phenotypes of SLX4IP^−/−^ cells. The combined loss of SLX4IP and SLX4 resulted in persistent PML-positive telomere aggregates, a robust G2/M arrest and synthetic growth defect in clonogenic survival assays, which we attribute to the onset of senescence. This finding strongly suggested that SLX4IP also conducts functions in ALT independent of SLX4.

A potential link between SLX4IP and the BLM helicase was suggested by the robust accumulation of BLM in PML-positive telomere clusters in SLX4IP^−/−^ cells depleted for SLX4 and by previous observations in *S. pombe* that telomere dysfunction in Taz1-deficient strains leads to telomere breakage and entanglement in a SUMOylated Rqh1-dependent manner (Rog et al., 2009). Indeed, we show that SLX4IP directly interacts with both BLM and SLX4 and is therefore ideally placed to influence the balance between recombination resolution and dissolution pathways.

Remarkably, we found that the synthetic growth defect caused by the combined loss of SLX4IP and SLX4 is rescued to near WT levels by depleting BLM but not by DNA2. In the absence of SLX4, we propose that intermediate processing shifts to BTR-dependent dissolution, but this is somehow constrained by the association between SLX4IP and BLM. However, in cells lacking both SLX4 and SLX4IP, resolution is compromised and BTR-dependent dissolution is unleashed, leading to pathological exacerbation of the ALT phenotype and synthetic growth arrest.

SLX4IP is dispensable for the endonuclease activities of the SMX complex, as recombinant SLX4, in combination with SLX1, MUS81, or XPF, is proficient for nucleolytic processing in the absence of SLX4IP *in vitro* (Wyatt et al., 2013, Wyatt et al., 2017). Similarly, loss of SLX4IP did not affect the E3 SUMO ligase activity of SLX4 (Figure S7L; Guervilly et al., 2015). Thus, SLX4IP is recruited to telomeres via its association with SLX4 but does not seem to be required for the core enzymatic functions of SLX4 and the SMX complex.

Because SLX4IP interacts directly with BLM, it is conceivable that SLX4IP directly regulates BLM activities. Although SLX4IP does not measurably inhibit BLM helicase activity *in vitro* (Figure S7M) or alters BLM protein stability, it is possible that SLX4IP affects the dissolution activity of the BTR complex or its ability to access the appropriate substrates. Our finding that SLX4IP interacts with XPF independently from SLX4 suggests an alternative scenario in which SLX4IP acts through the XPF-ERCC1 endonuclease to oppose BLM activity. Several observations support this hypothesis. First, SLX4 is dispensable for the interaction between XPF and SLX4IP. Second, we found that loss of XPF did not phenocopy SLX4 depletion in the context of SLX4IP deficiency, suggesting that SLX4IP and XPF might act in the same, SLX4-independent, pathway. Finally, previous reports have described SLX4-independent roles for XPF-ERCC1 in nucleotide excision repair and in the repair of topoisomerase inhibitor-induced DNA lesions (Fagbemi et al., 2011, Kim et al., 2013). Thus, SLX4IP may regulate XPF-ERCC1 at ALT telomeres and as such influence telomere length maintenance and counterbalance BLM.

Telomere maintenance in osteosarcomas frequently occurs via the ALT pathway, and we show here that SLX4IP is inactivated in a subset of these tumors. Intriguingly, loss of SLX4IP is potentially mutually exclusive with loss of ATRX, DAXX, and H3.3. However, because loss of SLX4IP is not sufficient to induce ALT-like phenotypes in ALT-negative cells, but its loss in ALT-positive cells augments telomere recombination, our data suggest that like ATRX, loss of SLX4IP may contribute to the establishment or maintenance of ALT in combination with additional insults. Chromosomal aberrations involving SLX4IP are also frequently found in acute lymphoblastic leukemia (Lilljebjörn et al., 2010, Meissner et al., 2014, Mullighan et al., 2007). Although leukemias are not generally associated with a positive ALT status (Heaphy et al., 2011b, Henson and Reddel, 2010), it will be important to test whether the subset of SLX4IP-deficient leukemias are ALT positive. Taken together, our findings raise the possibility that perturbing the balance between resolution and dissolution may provide new opportunities for therapeutic intervention in ALT-positive tumors, particularly those that harbor SLX4IP deficiency.

## STAR★Methods

### Key Resources Table

**Table:** 

REAGENT or RESOURCE	SOURCE	IDENTIFIER
**Antibodies**		

Mouse monoclonal anti-SLX4IP (clone G4)	Santa Cruz Biotechnology	Cat#sc-377066; RRID:AB_2752253
Sheep polyclonal anti-SLX4 (BTBD12, sheep S714C)	MRC PPU University of Dundee	Cat#DU16029; RRID:AB_2752254
Rabbit polyclonal anti-SLX4 (BTBD12)	Bethyl Laboratories	Cat#A302-270A, RRID:AB_1850156
Rabbit polyclonal anti-SLX1B (GIYD2)	Proteintech	Cat#21158-1-AP; RRID:AB_2752255
Mouse monoclonal anti-MUS81 (clone MTA30 2G10/3)	Abcam	Cat#ab14387; RRID:AB_301167
Mouse monoclonal anti-ERCC4 (XPF, clone 219)	Thermo Fisher Scientific	Cat#MA5-12054; RRID:AB_10981652
Mouse monoclonal anti-GFP (clones 7.1 and 13.1)	Roche	Cat#11814460001; RRID:AB_390913
Chicken polyclonal anti-GFP	Abcam	Cat#ab13970, RRID:AB_300798
Rabbit polyclonal anti-BLM	Abcam	Cat#ab2179; RRID:AB_2290411
Mouse polyclonal anti-ERCC6L (PICH)	Abcam	Cat# ab88560; RRID:AB_2041158
Mouse monoclonal anti-PML (clone PG-M3)	Santa Cruz Biotechnology	Cat#sc-966; RRID: RRID:AB_628162
Mouse monoclonal anti-RPA32 (clone 9H8)	Abcam	Cat#ab2175; RRID:AB_302873
Mouse monoclonal anti-γH2AX (clone JBW301)	Millipore	Cat#05-63; RRID:AB_309864
Rabbit polyclonal anti-γH2AX	Cell Signaling Technologies	Cat#2577; RRID:AB_2118010
Rabbit polyclonal anti-RAP1	Bethyl Laboratories	Cat#A300-306A; RRID:AB_162721
Rabbit polyclonal anti-DNA2	Abcam	Cat#ab96488, RRID:10677769
Mouse monoclonal anti-α-Tubulin	Sigma-Aldrich	Cat#T6074; RRID:AB_477582
Mouse monoclonal anti-Vinculin	Abcam	Cat#ab11194; RRID:AB_297835
Mouse monoclonal anti-SMARCAL1	Santa Cruz Biotechnology	Cat# sc-376377; RRID:AB_10987841
Rabbit monoclonal anti-ATRX	Santa Cruz Biotechnology	Cat# sc-15408; RRID:AB_2061023
Rabbit monoclonal anti-DAXX	Cell Signaling Technologies	Cat# 4533; RRID:AB_2088778
Mouse monoclonal anti-H3	Abcam	Cat# ab10799; RRID:AB_470239
Rabbit polyclonal anti-histone H3	Abcam	Cat# ab1791; RRID:AB_302613
Rabbit polyclonal anti-pH3 (Ser10)	Cell Signaling Technology	Cat# 9701, RRID:AB_331535
Mouse monoclonal anti-SUMO2/3 (clone 8A2)	Abcam	Cat#ab81371; RRID:AB_1658424
Mouse monoclonal anti-p62/SQSTM1 (clone 3)	BD Biosciences	Cat#610832; RRID:AB_398151
Mouse monoclonal anti-cytochrome C (clone 6H2.B4)	Thermo Fisher Scientific	Cat# 33-8200; RRID:AB_2533141
Goat polyclonal anti-mouse, horseradish peroxidase-conjugated	Dako	Cat#P0447; RRID:AB_2617137
Swine polyclonal anti-rabbit, horseradishperoxidase-conjugated	Dako	Cat#P0399; RRID:AB_2617141
Rabbit polyclonal anti-sheep, horseradishperoxidase-conjugated	Abcam	Cat#ab6747; RRID:AB_955453
Goat anti-chicken IgG (H+L), Alexa Fluor 488 conjugated	Invitrogen	Cat#A11039; RRID:AB_2534096
Goat anti-mouse IgG (H+L), Alexa Fluor 488 conjugated	Invitrogen	Cat#A11001; RRID:AB_2534069
Goat anti-rabbit IgG (H+L), Alexa Fluor 488 conjugated	Invitrogen	Cat#A11008; RRID:AB_143165
Goat anti-rabbit IgG (H+L), Alexa Fluor 546 conjugated	Invitrogen	Cat#A11010; RRID:AB_2534077
Sheep anti-digoxigenin-AP, Fab Fragments	Sigma	Cat#11093274910 RRID: AB_2734716

**Biological Samples**		

Osteosarcoma patient derived xenograft models	Pediatric Preclinical Testing Program; Houghton et al. Pediatric Blood and Cancer. 2007	PMID:17066459

**Chemicals, Peptides, and Recombinant Proteins**		

BrdU	Sigma-Aldrich	Cat#B5002
Camptothecin	Sigma-Aldrich	Cat#C9911
Mitomycin C	Sigma-Aldrich	Cat#M0503-5X2MG
Doxycycline	Sigma-Aldrich	Cat#D3447
Cycloheximide	Sigma-Aldrich	Cat#C4859
Blasticidin	ThermoFisher Scientific	Cat#A1113903
Hygromycin B	ThermoFisher Scientific	Cat#10687010
Mevinolin (lovastatin)	Sigma-Aldrich	Cat#M2147-25MG
RO-3306	Sigma-Aldrich	Cat#SML0569-25MG
Thymidine	Sigma-Aldrich	Cat#T1895-25G
Nocodazole	Sigma-Aldrich	Cat# M1404-2MG
Benzonase	Millipore	Cat#E1014-25KU
4x NuPAGE LDS sample buffer	Invitrogen	Cat#13778150
GFP-Trap_MA	Chromotek	Cat#gtma-20
ProLong Gold antifade with DAPI	Thermo Fisher Scientific	Cat#P36931
TAMRA-TelG 5′-(TTAGGG)3-3′ PNA probe	PNA Bio-synthesis	Cat#F1006
FITC-TelC 5′-(CCCTAA)3-3′ PNA probe	PNA Bio-synthesis	Cat#F1009
Phi29 DNA Polymerase	Thermo FisherScientific	Cat#EP0091
ATP, [γ-32P]- 6000Ci/mmol 10mCi/ml	Perkin Elmer	Cat#NEG502Z250UC
Blocking Reagent	Sigma-Aldrich	Cat#11096176001 ROCHE
Colcemid	Sigma-Aldrich	Cat#0295892001 ROCHE
EDTA-free Complete protease inhibitor cocktail	Roche	Cat#COEDTAF-RO
PhosSTOP phosphatase inhibitor cocktail	Roche	Cat#PHOSS-RO
Exonuclease III	Promega	Cat#M1815
Hoechst 33258	Sigma-Aldrich	Cat#861405
AluI	New England Biolabs	Cat#R0137
MboI	New England Biolabs	Cat#R0147
Phi-29 Polymerase	New England Biolabs	Cat#M0269
CDP-Star	Sigma Aldrich	Cat#11685627001
ULTRAhyb Ultrasensitive Hybridization Buffer	Thermo Fisher	Cat#AM8669
Dharmafect I Transfection Reagent	Dharmacon	Cat#T-2001-03
Lipofectamine RNAiMAX	Invitrogen	Cat#13778150
DIG Oligo 3′ End labeling kit (2^nd^ generation, Roche)	Sigma Aldrich	Cat#03353575910
QiaAMP DNA mini kit	QIAGEN	Cat#51304
RNeasy Mini Kit (250)	QIAGEN	Cat#74106
Kapa RNA HyperPrep kit with Riboerase	Kappa Biosystems	Cat#08098140702
QIAquick PCR purification kit	QIAGEN	Cat#28106
DIG Wash and Block Buffer Set	Sigma Aldrich	Cat# 11585762001
Senescence Cells Histochemical Staining Kit	GE Healthcare	Cat# CS0030-1KT
Human: U2OS	The Francis Crick Institute Cell Services	N/A
Human: WI38VA13	The Francis Crick Institute Cell Services	N/A
Human: HeLa 1.2.11	The Francis Crick Institute Cell Services	N/A
Human: HEK293	The Francis Crick Institute Cell Services	N/A
Human: RPE-1 hTERT	The Francis Crick Institute Cell Services	N/A
Human: U2OS FLP-IN HOST	Gift of Daniel Durocher	N/A
Human: U2OS FLP-IN GFP	This study	N/A
Human: U2OS FLP-IN GFP-SLX4IP WT	This study	N/A
Human: U2OS SLX4IP^−/−^ (clone 2) SLX4IP-pLenti-CMV-Blast-DEST	This study	N/A
Human: U2OS SLX4IP^−/−^ (clone 2) pLenti-CMV-Blast-DEST	This study	N/A
Human: HOS	Boston University	N/A
Human: HeLa	Boston University	N/A
Human: MG63	Boston University	N/A
Human: SJSA1	Boston University	N/A
Human: G292	Boston University	N/A
Human: SAOS2	Boston University	N/A
Human: HUO9	Boston University	N/A
Human: NOS1	Boston University	N/A
Human: NY	Boston University	N/A
Human: CAL72	Boston University	N/A
Human: CAL78	Boston University	N/A
Human: HUO3N1	Boston University	N/A
Human: hFOB1.19	Boston University	N/A

**Deposited Data**		

RNA sequencing data, GEO Series accession number GGSE124768	This study	Gene Expression Omnibus/NCBI GEO: GSE124768https://www.ncbi.nlm.nih.gov/geo/query/acc.cgi?acc=GSE124768

**Experimental Models: Organisms/Strains**		

Mouse: CB17SC-F *scid*^−/−^ female mice	Taconic	CB17SC-F RF

**Oligonucleotides**		

ON-TARGET plus Non-targeting Pool	Dharmacon	D-001810-10
ON-TARGET plus SMARTpool human SLX4	Dharmacon	L-014895-00
ON-TARGET plus SMARTpool human SLX1A	Dharmacon	L-034933-01
ON-TARGET plus SMARTpool human MUS81	Dharmacon	L-016143-01
ON-TARGET plus SMARTpool human ERCC4	Dharmacon	L-019946-00
ON-TARGET plus SMARTpool human BLM	Dharmacon	L-007287-00
ON-TARGET plus SMARTpool human DNA2	Dharmacon	L-026431-01
siGENOME SMARTpool human TRF2	Dharmacon	M-003546-00
TelG probe (TTAGGG)_4_	This study	N/A
TelC probe (CCCTAA)_4_	This study	N/A
Alu probe 5′-GTAATCCCAGCACTTTGG-3′	This study	N/A

**Recombinant DNA**		

EGFP-C1-GFP-BLM	Addgene	Cat#80070;RRID:Addgene_80070
pcDNA5-FRT/TO-GFP	Gift from Daniel Durocher	DD982
pcDNA5-FRT/TO-GFP-SLX4-FL	Gift from John Rouse, Wilson et al., 2013	PMID:23994477
pcDNA5-FRT/TO-GFP-SLX4-WT (1-669)	This study	N/A
pcDNA5-FRT/TO-GFP-SLX4-A (1-200)	This study	N/A
pcDNA5-FRT/TO-GFP-SLX4-B (201-400)	This study	N/A
pcDNA5-FRT/TO-GFP-SLX4-C (401-669)	This study	N/A
pcDNA5-FRT/TO-GFP-SLX4-MLR (409-555)	This study	N/A
pET-SUMO-SLX4IP	This study	N/A
pcDNA5-FRT/TO-GFP-SLX4IP-FL	This study	N/A
pcDNA5-FRT/TO-GFP-SLX4IP-A (1-120)	This study	N/A
pcDNA5-FRT/TO-GFP-SLX4IP-B (121-230)	This study	N/A
pcDNA5-FRT/TO-GFP-SLX4IP-C (231-408)	This study	N/A
pcDNA5-FRT/TO-GFP-SLX4IP-ΔA (121-408)	This study	N/A
pcDNA5-FRT/TO-GFP-SLX4IP-ΔB (Δ121-230)	This study	N/A
pcDNA5-FRT/TO-GFP-SLX4IP-ΔC (1-231)	This study	N/A
px335-U6-Chimeric_BB-CBh-hSpCas9n(D10A)	Addgene	Cat#:42335;RRID:Addgene_42335
px335-C20A	This study	N/A
px335-C20B	This study	N/A
pLentiCRISPRv2	Addgene	Cat#:52961;RRID:Addgene_52961
pLentiCRISPRv2_SLX4IP_A	This study	N/A
pLentiCRISPRv2_SLX4IP_B	This study	N/A
pLenti-CMV-Blast-DEST	Addgene	Cat#17451; RRID:Addgene_17451
SLX4IP-pLenti-CMV-Blast	This study	N/A
His-SUMO3	Guervilly et al., 2015	PMID:25533188
FHA-SLX4 WT	Guervilly et al., 2015	PMID:25533188
FHA-SLX4 SIM^∗^	Guervilly et al., 2015	PMID:25533188

**Software and Algorithms**		

Adobe Photoshop CS5.1	Adobe	http://www.adobe.com/es/products/photoshop.html
Prism 7	GraphPad Software	https://www.graphpad.com/
Fiji	NIH	https://imagej.net/Fiji/Downloads
Volocity 6.3	PerkinElmer	http://cellularimaging.perkinelmer.com/downloads/detail.php?id=14
FV10-ASW 4.2	Olympus	https://www.olympus-lifescience.com/en/support/downloads/#!dlOpen=%23detail847249651
FV31S-SW	Olympus	https://www.olympus-lifescience.com/en/support/downloads/
Cell Profiler	Broad Institute	http://cellprofiler.org/releases/
Image Lab 5.2.1	Bio-Rad Laboratories	http://www.bio-rad.com/en-uk/product/image-lab-software?ID=KRE6P5E8Z
FlowJo v10	FlowJo	https://www.flowjo.com/solutions/flowjo/downloads
FastQC	N/A	https://www.bioinformatics.babraham.ac.uk/projects/fastqc/
Salmon	N/A	https://github.com/COMBINE-lab/Salmon
ggplot2 package	N/A	https://cran.r-project.org/web/packages/ggplot2/index.html
GXCapture	GT Vision	https://www.gtvision.co.uk/GX-Capture-Camera-Control-Image-Capture-Storage-Annotation-Enhancement-Analysis-FREE

**Other**		

SLX4IP CRISPR target sequence C20A 5′-GATCTTCATATCTTGCCACA AGG-3′	This study	N/A
SLX4IP CRISPR target sequence C20B 5′-CCA TTAATGTCTTTCAGTGTGGG-3′	This study	N/A
SLX4IP CRISPR target sequence SLX4IPA 5′-GATCTTCATATCTTGCCACA-3′	This study	N/A
SLX4IP CRISPR target sequence SLX4IPB 5′-TGGGAATTTTGCTGTCCTCG-3′	This study	N/A

### Lead Contact and Materials Availability

Further information and requests for resources and reagents should be directed to the Lead Contact, Simon Boulton (simon.boulton@crick.ac.uk).

### Experimental Model and Subject Details

#### Cell lines

At the Francis Crick Institute, the following human cell lines were used: U2OS (female), WI38VA13 (female), HeLa 1.2.11 (female), HEK293 (female), RPE -1 hTERT (female), U2OS FLP-IN HOST, U2OS FLP-IN GFP, U2OS FLP-IN GFP-SLX4IP WT, U2OS SLX4IP^−/−^ clone 1, U2OS SLX4IP^−/−^ clone 2, WI38VA13 SLX4IP^−/−^, HeLa 1.2.11 SLX4IP^−/−^, HEK293 SLX4IP^−/−^, RPE1 h-TERT SLX4IP^−/−^, U2OS SLX4IP^−/−^ (clone 2) SLX4IP-pLenti-CMV-Blast-DEST and U2OS SLX4IP^−/−^ (clone 2) pLenti-CMV-Blast-DEST. All host cell lines (U2OS, WI38VA13, HeLa 1.2.11, RPE-1 hTERT) were authenticated by Francis Crick Institute Cell Services. Cells were cultured in an environmental incubator set to 37°C and 5% CO2 and were maintained using standard tissue culture procedures. All cell lines were cultured in DMEM supplemented with 10% fetal bovine serum (FBS). Cells were frozen in 10% FBS/5% DMSO/ medium using Mr. Frosty freezing containers (Nalgene) according to the manufacturer’s instruction. For long-term storage, cells were kept in a liquid nitrogen tank. The inducible GFP-SLX4IP cell lines were generated using the Flp-In T-REx system (Invitrogen) as described in the manufacturer’s protocol. Each construct was cloned into the pcDNA5-FRT-TO-GFP vector followed by co-transfection with the pOG44 vector (Flp recombinase) into U2OS host cell lines. The host cell line was cultured in DMEM supplemented with 15.5 μg/ml zeocin (Invitrogen) and 4 μg/ml blasticidin (Invitrogen). Recombination events were selected with 250 μg/ml hygromycin B (ThermoScientific). Flp-In T-REx stable cell lines were cultured in DMEM supplemented with 5 μg/ml blasticidin and 250 μg/ml hygromycin B. For the cycloheximide chase, 20 μg/ ml cycloheximide (Sigma, C4859) was added to the medium for the indicated time points.

At Boston University, the following human cell lines were used: HOS, HeLa, MG63, SJSA1, G292, SAOS2, HUO9, NOS1, NY, CAL72, CAL78, HUO3N1 and hFOB1.19. G292, SJSA1 CAL78 and HUO3N1 were cultured in RPMI 1640, 10% FBS, 1% Sodium Pyruvate and 1% Penicillin/Streptomycin. HOS were cultured in Eagle’s Minimum Essential Medium, 10% FBS, 1% Pencillin/Streptomycin. HUO9 and NOS1 were cultured in RPMI 1640 5% FBS, 1% Sodium Pyruvate and 1% Penicillin/Streptomycin. NY and MG63 were cultured in DMEM/F12, 5% FBS, 1% Penicillin/Streptomycin. CAL72 were cultured in DMEM/F12, 10% FBS, 1% Penicillin/Streptomycin. U2OS were cultured in DMEM, 10% FBS, 1% Penicillin/Streptomycin. SAOS2 were cultured in RPMI 1640, 10% FBS, 1% Penicillin/Streptomycin. All cells were maintained at 37°C in a humidified incubator with 5% CO2 except for hFOB1.19. hFOB1.19 were cultured in phenol red free DMEM/F12, with 10% FBS, 2.5 mM L-glutamine, 0.3 mg/ml G418. hFOB1.19 were maintained at 34°C in a humidified incubator with 5% CO2.

#### PDX Xenografts

Early passage, viably frozen patient derived xenograft tumor sections were obtained courtesy of Dr. Peter Houghton and the Pediatric Preclinical Testing program (Houghton et al., 2007). CB17SC-F *scid*^−/−^ female mice (Taconic) were used to propagate subcutaneous implanted tumors fragments. Mice were maintained in sterile cages under barrier conditions using protocols and conditions approved by the institutional animal care and use committee. Following tumor engraftment and growth, PDX tissue was harvested, flash frozen, and stored at −80°C.

### Method Details

#### Cloning and CRISPR

All DNA preparations (including PCR clean-up, agarose gel extractions, minipreps and maxipreps) were done with DNA purification kits from QIAGEN according to the manufacturer’s instructions. Internal deletions and point mutations were generated by Quikchange (Stratagene). All constructs were confirmed by sequencing. The cDNA for human *SLX4IP* (4840139 (IMAGE ID), IRALp962P0138Q sequence verified, purchased from Source Bioscience) was amplified by PCR and ligated into pcDNA5-FRT/TO-GFP (a kind gift from Daniel Durocher) and pET-SUMO (Invitrogen). To generate SLX4IP-A, a fragment encompassing amino acid residues 1-120 was PCR-amplified and ligated into pcDNA5-FRT/TO-GFP. To generate SLX4IP-B, a fragment encompassing amino acid residues 121-230 was PCR-amplified and ligated into pcDNA5-FRT/TO-GFP. To generate SLX4IP-C, a fragment encompassing amino acid residues 231-408 was PCR-amplified and ligated into pcDNA5-FRT/TO-GFP. To generate SLX4IP-ΔA, a fragment encompassing amino acid residues 121-408 was PCR-amplified and ligated into pcDNA5-FRT/TO-GFP. To generate SLX4IP-ΔC, a fragment encompassing amino acid residues 1-231 was PCR-amplified and ligated into pcDNA5-FRT/TO-GFP. The details of the internal pcDNA5-FRT/TO-GFP-SLX4IP deletions and point mutations are as follows: SLX4IP-ΔB, amino acid residues Δ121-230; SLX4IP L16K/V17K, mutates SIM1; SLX4IP V115K/V116K, mutates SIM2. The pcDNA5-FRT/TO-GFP-SLX4 expression plasmid was a kind gift from John Rouse. To generate SLX4-WT, a fragment encompassing amino acid residues 1-669 was PCR-amplified and ligated into pcDNA5-FRT/TO-GFP. To generate SLX4-A, a fragment encompassing amino acid residues 1-200 was PCR-amplified and ligated into pcDNA5-FRT/TO-GFP. To generate SLX4-B, a fragment encompassing amino acid residues 201-400 was PCR-amplified and ligated into pcDNA5-FRT/TO-GFP. To generate SLX4-C, a fragment encompassing amino acid residues 401-669 was PCR-amplified and ligated into pcDNA5-FRT/TO-GFP. To generate SLX4-MLR, a fragment encompassing amino acid residues 409-555 was PCR-amplified and ligated into pcDNA5-FRT/TO-GFP. The expression plasmids used for the *in vivo* sumoylation assay were described in Guervilly et al., 2015. The EGFP-C1-GFP-BLM expression plasmid was obtained from Addgene (Cat#80070; RRID:Addgene_80070). Control and SLX4IP complimented cells were generated by transducing SLX4IP^−/−^ clone #2 cells with virus produced from empty pLenti-CMV-Blast-DEST (control) and SLX4IP-pLenti-CMV-Blast (SLX4IP), respectively. Cells were then selected in 10ug/ml blasticidin.

*SLX4IP* knockout cells were generated essentially as described in Sanjana et al. (2014). The sgRNAs were designed with the CRISPR Design Tool from Genome Engineering (http://tools.genome-engineering.org). To knock out *SLX4IP* in U2OS and WI38VA13 two CRISPR guide RNAs (denoted as C20A and C20B) were cloned into px335-U6-Chimeric_BB-CBh-hSpCas9n(D10A) (obtained from Addgene, Cat#42335; RRID:Addgene_42335). The guide RNAs target the following sequences: C20A, 5′- GATCTTCATATCTTGCCACAAGG-3′; C20B, 5′-CCA TTAATGTCTTTCAGTGTGGG-3′. px335-C20A and px335-C20B were co-transfected into the host cell lines and single cell clones were isolated. To knock out *SLX4IP* in HeLa 1.2.11 cells, a single guide RNA targeting the following sequence was cloned into pLentiCRISPRv2: 5′-GATCTTCATATCTTGCCACA-3′ (denoted as SLX4IPA). To knock out *SLX4IP* in HEK293 and RPE1 hTERT cells, a single guide RNA targeting the following sequence was cloned into pLentiCRISPRv2: 5′-TGGGAATTTTGCTGTCCTCG-3′ (denoted as SLX4IPB). The single guide RNA plasmids, together with ViraPower viral packaging plasmids (Invitrogen), were transfected into 293FT cells using Lipofectamine 2000 (Invitrogen) according to the manufacturer’s protocol. Lentiviral supernatants were collected 72 h after transfection, filtered through a 0.45-μm filter, and used for spin transduction of HeLa 1.2.11, RPE1-hTERT and HEK293 cells. Transduced cells were selected with 1 μg/ml puromycin for 72 h after transduction. After lentiviral infection, single cell clones were isolated. Knockouts were confirmed by SLX4IP immunoblotting and sequencing.

#### Plasmid transfections and RNA interference

Plasmid transfections were carried out using either the Effectene Transfection Reagent (QIAGEN) or Lipofectamine 2000 (Invitrogen) following the manufacturers protocols. RNAi transfections were performed using either Dharmafect 1 (ThermoFisher) or Lipofectamine RNAiMAX (Invitrogen) in a forward transfection mode following the manufacturers protocols. 5 hours after transfection, the medium was substituted for fresh medium. Cells were generally collected 24 hours after plasmid transfection and 72 hours after RNAi transfection.

#### Laser damage

Cells were seeded on 8 well Lab-Tek chamber slides (Thermo Fisher Scientific). Cells were pre-sensitized with 10 μM BrdU and treated with 1 μg/ml doxycycline 24 hours prior to imaging. Cells were transferred to an Olympus FV3000 confocal laser-scanning microscope with a heat and atmosphere controlled incubator. Laser micro-irradiation was performed with a 405 nm laser focused through a 60x objective. To ensure that cells with similar expression levels are assayed and that GFP stayed within the dynamic detection range, cells exhibiting moderate expression levels were systematically chosen using identical 488 nm laser settings.

#### Indirect immunofluorescence

Cells were grown on #1.5 glass coverslips. Cells were fixed with 2% (w/v) formaldehyde (Thermo Scientific) in PBS for 20 min at room temperature. After fixation, cells were washed with 1X PBS four times and then blocked with ADB (Antibody Dilution Buffer; 10% normal goat serum, 0.1% Triton X-100, 0.1% saponin in PBS) for 30 min. Cells were incubated with primary antibody (diluted in ADB) for 1 hour at room temperature, washed three times with 1X PBS and then counterstained with Alexa Fluor 488 goat anti-mouse IgG and Alexa Fluor 546 goat anti-rabbit IgG secondary antibodies (Molecular Probes) diluted in ADB, for 1 hour at room temperature. Cells were then washed three times with 1X PBS. The coverslips were mounted onto glass slides with Prolong Gold mounting agent supplemented with DAPI (Life Technologies). Images were acquired with an Olympus FLV1000 inverted microscope equipped with a 63X oil objective. Following acquisition, images were imported into ImageJ (NIH) and Adobe Photoshop CS5 for manual quantitation.

#### Telomeric Peptide Nucleic Acid Fluorescence *In Situ* Hybridization (PNA-FISH)

Cells were treated with 0.2 μg/ml of colcemid for 90 minutes to arrest cells in metaphase. Trypsinized cells were then incubated in 75 mM KCl for 20 min and pelleted at 1000rpm for 5 min, fixed with methanol:acetic acid (3:1), spread on glass slides and left overnight at room temperature to dry. The slides were rehydrated in PBS for 5 minutes, fixed in 4% formaldehyde for 5 minutes, treated with 1 mg/ml of pepsin for 10 minutes at 37°C, and fixed in 4% formaldehyde for 5 minutes. Next, slides were dehydrated in 70%, 85%, and 100% (v/v) ethanol for 15 minutes each and then air-dried. Metaphase chromosome spreads were hybridized with a telomeric TAMRA-TelG 5′-(TTAGGG)_3_-3′ PNA probe (Bio-synthesis) in hybridizing solution (70% formamide, 0.5% blocking reagent (Roche), 10mM Tris-HCl pH 7.2) for 90 s at 80°C followed by 2 hours at room temperature and washed twice with washing buffer (70% formamide, 10mM Tris-HCl pH 7.2) for 15 min at room temperature. Slides were mounted using ProLong Gold antifade with DAPI (Life Technologies). Chromosome images and telomere signals were captured using Zeiss Axio Imager M1 microscope equipped with an ORCA-ER camera (Hamamatsu) controlled by Volocity 6.3 software (Improvision). For quantitative FISH (Q-FISH) analysis, the telomere fluorescence distribution of individual telomere dots was quantified using Cell Profiler (Broad Institute).

#### Immunofluorescence coupled to fluorescence *in situ* hybridization (IF-FISH)

Cells were grown on #1.5 glass coverslips. Cells were fixed with 2% (w/v) formaldehyde (Thermo Scientific) in PBS for 20 min at room temperature. After fixation, cells were washed with 1X PBS four times and then blocked with ADB (Antibody Dilution Buffer; 10% normal goat serum, 0.1% Triton X-100, 0.1% saponin in PBS) for 30 min. Cells were incubated with primary antibody (diluted in ADB) for 1 hour at room temperature, washed three times with 1X PBS and then counterstained with Alexa Fluor secondary antibodies (Molecular Probes) diluted in ADB, for 1 hour at room temperature. Cells were washed three times with 1X PBS, fixed again with 2% (w/v) formaldehyde in PBS for 20 min at room temperature and then washed twice with 1X PBS. Next, coverslips were dehydrated in 70%, 85%, and 100% (v/v) ethanol for 5 minutes each and then air-dried. Dry coverslips were hybridized with a telomeric TAMRA-TelG 5′-(TTAGGG)_3_-3′ PNA probe (Bio-synthesis) in hybridizing solution (70% formamide, 0.5% blocking reagent (Roche), 10mM Tris-HCl pH 7.2) for 90 s at 80°C followed by 2 hours at room temperature and washed twice with washing buffer (70% formamide, 10mM Tris-HCl pH 7.2) for 15 min at room temperature. The coverslips were mounted onto glass slides with Prolong Gold mounting agent supplemented with DAPI (Life Technologies). Images were acquired with an Olympus FLV1000 inverted microscope equipped with a 63X oil objective. For each image, Z sections (0.2 μm apart) were acquired with 3 signal channels. Following acquisition, images were imported into Fiji (NIH) and Adobe Photoshop CS5.1 for manual quantitation. The analysis of fluorescence intensities presented in Figure 1D was performed on TIFF images using Fiji (NIH). A straight line in a single Z section was drawn through the nucleus, along which fluorescence intensities were measured. APB size was quantified using Cell Profiler (Broad Institute). In all micrographs dashed lines indicated nucleus outlines (as determined by DAPI staining); insets represent 3 X magnifications of the indicated fields.

#### Detection of telomere synthesis

Cells were grown on #1.5 glass coverslips. To detect telomeric DNA synthesis 100 μM EdU (Thermo Fisher Scientific) was added for 2 hours to the medium prior to fixation with 2% (w/v) formaldehyde (Thermo Scientific) in PBS for 20 min at room temperature. EdU incorporation was visualized using the Click-iT Plus EdU Alexa Fluor 488 Imaging Kit according to manufacturer’s instructions. Following the Click-iT reaction, the cells were fixed again with 2% (w/v) formaldehyde in PBS for 20 min at room temperature and washed twice with 1X PBS. Next, coverslips were dehydrated in 70%, 85%, and 100% (v/v) ethanol for 5 minutes each and air-dried. Dry coverslips were hybridized with a telomeric TAMRA-TelG 5′-(TTAGGG)_3_-3′ PNA probe (Bio-synthesis) and mounted on glass slides as described above.

#### Chromosome-orientation fluorescence *in situ* hybridization (CO-FISH)

Cells were incubated with 10 μM BrdU for 20 hours and were then with treated with 0.2 μg/ml of colcemid for 90 minutes to arrest cells in metaphase. Trypsinized cells were incubated in 75 mM KCl for 20 min and pelleted at 1000rpm for 5 min, fixed with methanol:acetic acid (3:1), spread on glass slides and left overnight at room temperature to dry. The slides were rehydrated in PBS for 5 minutes, treated with 0.5mg/ml RNaseA (in PBS) for 15 minutes at 37°C and then stained with 0.5 μg/ml Hoechst 33258 (Sigma, in 2X SSC) for 20 minutes at room temperature. Next, the slides were places in a shallow plastic tray, covered with a thin layer of 2X SSC and exposed to 365 nm UV (Stratalinker 1800 UV irradiator) for 45 minutes at room temperature. The BrdU-labeled strand was then digested with 10 U/μl Exonuclease III (Promega) in the buffer supplied by the manufacturer for 20 min at room temperature. The slides were washed once in 1X PBS for 5 minutes, dehydrated in 70%, 85%, and 100% (v/v) ethanol for 5 minutes each and then air-dried. Metaphase chromosome spreads were hybridized with a telomeric TAMRA-TelG 5′-(TTAGGG)_3_-3′ PNA probe (Bio-synthesis) in hybridizing solution (70% formamide, 0.5% blocking reagent (Roche), 10mM Tris-HCl pH 7.2) for 2 hours at room temperature and rinsed once with wash buffer I (70% formamide, 10mM Tris-HCl pH 7.2, 0.1% (w/v) BSA). The slides were then hybridized with a telomeric FITC-TelC 5′-(CCCTAA)_3_-3′ PNA probe (Bio-synthesis) in hybridizing solution (70% formamide, 0.5% blocking reagent (Roche), 10mM Tris-HCl pH 7.2) for another 2 hours at room temperature, washed twice with wash buffer I for 15 minutes at room temperature and washed three times with wash buffer II (0.1M Tris-HCl pH 7.2, 0.15M NaCl, 0.08% (v/v) Tween-20) for 5 minutes at room temperature. Slides were mounted using ProLong Gold antifade with DAPI (Life Technologies). Chromosome images and telomere signals were captured using Zeiss Axio Imager M1 microscope equipped with an ORCA-ER camera (Hamamatsu) controlled by Volocity 6.3 software (Improvision).

#### Whole-cell extracts

Cells were rinsed with 1X PBS, trypsinized and collected in DMEM. Cells were pelleted by centrifugation at 500 g for 5 min and washed once more with 1X PBS. Cell pellets were frozen on dry ice and stored at −80°C. For lysis, cell pellets were thawed on ice, resuspended in 50 mM HEPES-KOH, pH 7.5, 100 mM KCl, 2mM EDTA, 0.5% IGEPAL CA-630, 10% glycerol, 1mM DTT, 1X protease inhibitors (Complete, EDTA-free, Roche) and 1X Phos-Stop (Roche)), incubated on ice for 30 min and gently syringed with a 23G needle. Cell lysates were clarified by centrifugation at 13 000 g for 20 min at 4°C. Protein concentration was determined using the BCA method (DC protein assay (Biorad)) according to the manufacturer’s instructions. Lysates were denatured in 2X NuPAGE LDS sample buffer (Invitrogen) for 5 min at 100°C, frozen on dry ice and stored at −80°C.

#### SDS-PAGE and immunoblotting

Proteins were separated by SDS-PAGE using NuPAGE mini gels (Invitrogen) and transferred onto a PVDF membrane (Millipore, Immobilon-P) using standard procedures. After transfer, the membrane was blocked in 5% skim milk/ TBST (TBS/ 0.1% Tween-20) for 30 min at room temperature and incubated with the indicated primary antibody (diluted in 5% skim milk/ TBST) for overnight at 4°C. The membrane was then washed 5 times for 5 min with TBST, incubated with a horseradish peroxidase-conjugated secondary antibody for 1 h at room temperature, and washed again 5 times for 5 min with TBST. The immunoblot was developed using ECL Western Blotting Reagent (Sigma) or SuperSignal West Femto (Thermo Fisher Scientific). All incubations were carried out on a horizontal shaker.

#### T-circle assay

To isolate genomic DNA, cells were then resuspended in TNE (10 mM Tris pH 7.4, 10 mM EDTA, 100 mM NaCl) and lysed in TNES (10 mM Tris pH 7.4, 100 mM NaCl, 10 mM EDTA, 1% SDS) in the presence of 100 μg/ml proteinase K. After overnight incubation with proteinase K at 37°C, and phenol/chloroform extractions, DNA was precipitated with isopropanol and resuspended in TE (10 mM Tris pH 7.5/1 mM EDTA). RNase A treatment, phenol/chloroform extractions and isopropanol precipitation followed. 3 μg of genomic DNA was digested with AluI/Hinf1, ethanol-precipitated and resuspended in an annealing buffer (0.2 M Tris [pH 7.5], 0.2 M KCl, and 1 mM EDTA) with 1 μM (TTAGGG)_4_ primer containing thiophosphate linkages between the three 3′ terminal nucleotides. The mix was denatured at 96°C for 5 min and cooled down to 25°C for 1 hour. DNA was ethanol precipitated and resuspended in 20 μL of the TCA reaction buffer (33 mM Tris-acetate [pH 7.9], 10 mM magnesium acetate, 66 mM potassium acetate, 0.1% Tween 20, 1 mM DTT, and 0.37 mM dNTPs). Primer extension was carried out with 7.5 U of Phi29 DNA polymerase (Thermo Scientific) at 30°C for 12 hours. Phi29 DNA polymerase was inactivated by incubation at 65°C for 20 min. The extension products were separated by denaturing gel electrophoresis (0.8% agarose, 50 mM NaOH, and 1 mM EDTA [pH 8]) at 2 V/cm for 18 hours and transferred onto a nylon membrane (GE Healthcare) in 10X SSC. The membrane was UV crosslinked and hybridized with a γ[32P]-labeled (TTAGGG)_4_ telomeric probe. Southern blot images were captured with a Storm 840 or an Odyssey CLx scanner. T-circle levels were quantified in ImageJ and were normalized to control reactions lacking Phi29 polymerase.

#### C-circle Assay

The c-circle assays in Figures S4 and S5 were done as follows: The C-circle assay protocol was adapted from Henson et al. (2017). Genomic DNA was extracted by incubating cells with 50 μl of QCP lysis buffer (50 mM KCl, 10 mM Tris-HCl pH 8.5, 2 mM MgCl_2_, 0.5% IGEPAL CA-630, 0.5% Tween-20) and 3 μl of QIAGEN protease shaking at 1,400 rpm at 56°C for 1 hour. The QIAGEN protease was inactivated by incubating the samples at 70°C for 20 min. DNA concentration was measured by fluorimetry using the Qubit dsDNA HS Assay (Thermo Fisher Scientific). Samples purified from ALT+ cells (U2OS and VA-13) were pre-diluted in QCP lysis buffer at 5 ng/μl, whereas samples purified from ALT- cells (HEK293, HeLa 1.2.11 and RPE-1 hTERT) were pre-diluted in QCP lysis buffer at 30 ng/μl. 5 ng (ALT+) or 30 ng (ALT-) of DNA were diluted to 10 μl in 10mM Tris-HCl pH 7.6 and mixed with 9.25 μl of Rolling Circle Master Mix (RCMM) (8.65mM DTT, 2.16X 10X φ29 Buffer, 8.65μg/mL BSA, 0.216% Tween-20 and 2.16mM of each dATP, dCTP, dGTP and dTTP) and 0.75 μl of φ29 DNA Polymerase (Thermo Fisher Scientific). Rolling Circle Amplification was performed by incubating samples in a thermocycler at 30°C for 8 hours, polymerase was inactivated at 70°C for 20 min and then kept at 8-10°C. For slot blot detection, samples were blotted onto Amersham Hybond N+ positively charged nylon membranes (GE Healthcare) under native conditions. After crosslinking, membranes were hybridized with γ-32P labeled Tel-C oligo probe (CCCTAA)_4_ in hybridization buffer (1.5X SSPE, 10% polyethylene glycol (PEG) MW 8000, 7% SDS) for 16h at 50°C. Membranes were exposed onto a phosphorimaging plate (GE Healthcare) and scanned using Typhoon FLA 9500 (GE Healthcare). Membranes were stripped in wash solution (0.5X SSC, 0.1% SDS) at 65°C and re-hybridized with γ-32P labeled Alu oligo probe 5′-GTAATCCCAGCACTTTGG-3′ for 16h at 37°C as a loading control.

The c-circle assays shown in Figure 7 were done as follows. The c-circle assay was performed as previously described (Henson et al., 2009). Briefly, genomic DNA was isolated from 25-50 mg of frozen tumor tissue using the QIAGEN QiaAMP DNA Mini Kit according to the manufacturer’s instructions. Following purification, genomic DNA was digested with AluI and MboI restriction enzymes overnight at 37°C, and then purified using a QIAGEN PCR clean-up kit according to the manufacturer’s instructions. Purified, digested DNA was quantified with a Nanodrop spectrophotometer and then diluted to a concentration of 10 ng/μl. gDNA was diluted in 25 μl of 1X Φ29 buffer (NEB) containing BSA (NEB; 0.08 mg/ml), 0.1% Tween-20, 0.25 mM each of dATP, dGTP, and dTTP, then incubated in the presence or absence of 7.5 U Φ29 polymerase (NEB) at 30°C for 8 hours, then 65°C for 20 minutes. 80 ng of gDNA was incubated in the presence of Φ29 polymerase, and 20 ng of gDNA was incubated in the absence of Φ29 polymerase. Amplification products were diluted to 10X SSC and run through a dot blot apparatus onto a Hybond N+ membrande using a BioRad dot blot vacuum manifold. The membrane was crosslinked for 35 s (125J). The membrane was incubated in Ultra-Hyb hybridization buffer (Ambion) for 1 hour at 50°C. Telomeric probe (CCCTAA)_4_ was labeled using the DIG oligonucleotide 3ʹ- end labeling kit (2nd generation, Roche) according to manufacturer’s instructions. DIG labeled probe was added to the Ultra-Hyb hybridization buffer (1:1000) and incubated overnight at 50°C. The following day, the membrane was washed twice with 2X SSC + 0.1% SDS at room temperature for 5 minutes each and twice with 0.5X SSC + 0.1% SDS at 50°C for 15 minutes each. The membrane was developed using anti-DIG-AP (Roche), CDP-star (Roche), and the DIG Wash and Block Buffer set (Roche) following manufacturer’s instructions. C-circles were quantified using densitometry, first subtracting the signal from the no polymerase control, and then normalizing to the negative control (SJSA1, non-ALT).

#### Clonogenic survival assay

Cells transfected were trypsinized, counted and re-plated into 6-well dishes. Each condition was plated in duplicate. Cells were grown for 9-11 days and fixed in a 20% (v/v) methanol/0.4% (w/v) crystal violet solution for 5 min.

#### Fluorescence-activated cell sorting (FACS)

Cells were trypsinised and fixed in 70% ethanol. Cells were then resuspended in an RNase A (20 μg/ml) and propidium iodide (50 μg/ml) solution, passed through a 70 μm cell strainer and the cell cycle distribution of the cells analyzed by flow cytometry, using a 610/20 gate. Gating and analysis was performed manually using FlowJo v10 (FlowJo).

#### Senescence-associated β-galactosidase staining

U2OS cells were re-seeded into 6 well plates at a density of 300 cells/ well 72 hours post-siRNA transfection. Cells were then incubated for 11 days and processed for β-galactosidase staining using a Senescence Cells Histochemical Staining Kit (GE Healthcare, CS0030-1KT) according to the manufacturer’s instructions. Cells were imaged with a an Olympus CKX41 microscope using a GXCAM-H5 camera and GXCapture software.

#### Quantitative RT-PCR

RNA was first isolated using the RNeasy Mini Kit (QIAGEN) and then reverse transcribed using the High-Capacity RNA-to-cDNA kit (Thermo Fisher Scientific) according to the manufacturers’ instructions. RT-qPCR was performed with the following primers: BLM TaqMan probe_Hs00172060_m1 (Cat# 4331182, ThermoFisher) and GAPDH TaqMan probe_Hs02758991_g1 (Cat# 4448484, ThermoFisher) using the SsoAdvanced Universal Supermix (Biorad).

#### *In vivo* SUMOylation

One million U2OS cells were seeded in 60 mm dishes, transfected with 5μg of plasmid DNA (3.5 μg His-SUMO3 + 1.5 μg of control or SLX4 expression vector) and treated with 500 ng/ml Doxycycline (Sigma) to induce exogenous SLX4 expression. Cells were collected 24 hours later and cell pellets were frozen at −80°C for at least one night. Cell pellets were lysed in 400μL of denaturing urea buffer (8 M Urea, 115 mM NaH_2_PO_4_, 300 mM NaCl, 10 mM Tris-HCl [pH = 8.0], 0.1% [v/v] NP-40, 5 mM Imidazole) for 1 hour at room temperature. Extracts were incubated with TALON metal affinity resin (Clontech) for 1 hour at room temperature. Beads were washed 3 times with urea buffer before elution in loading buffer supplemented with 30mM EDTA.

#### Immunoprecipitation

Cells were first washed with ice-cold 1xPBS and scraped from the dish in a Lysis buffer (50 mM HEPES-KOH, pH 8.0; 100 mM KCl; 2 mM EDTA; 0.5% Nonidet P-40 substitute; 10% glycerol; phosSTOP (Sigma-Aldrich); cOmplete, Mini, EDTA-free Protease Inhibitor Cocktail (Sigma-Aldrich); 1 mM DTT). Lysates were then syringed 6 times using a 23G needle and clarified by centrifugation at 13 000 x g for 30 min at 4°C. Protein concentration was determined using the BCA method (DC protein assay (Biorad)) according to the manufacturer’s instructions. The input sample was prepared by adding 4x NuPAGE LDS sample buffer supplemented with 2-mercaptoethanol (final concentration 89.3 mM) and boiled for 10 minutes at 95°C. For co-immunoprecipitation, GFP-Trap_MA resin was washed three times with Lysis buffer. The lysates were then added on the washed resin and incubated for 2 hours at 4°C on a rotating wheel. Resin with bound proteins was then washed 4 times with ice-cold Lysis buffer. The immunoprecipitated proteins were eluted by resuspending the beads in 1x NuPAGE LDS sample buffer (with 89.3 mM 2-mercaptoethanol) and boiling for 10 minutes at 95°C. Eluates were separated from magnetic beads and transferred into a new tube, before freezing at −80°C until immunoblotting analysis.

In Figure S7D, after syringing of the lysates benzonase was added to the respective lysate made with Lysis buffer (without EDTA, with added 10 mM magnesium chloride) in the concentration 1000 U/ml and the lysates were incubated in the cold room on a rotating wheel for 1 hour and 45 minutes after which they were clarified by centrifugation, followed by immunoprecipitation protocol described above.

#### Cell cycle synchronization

U2OS Flp-In T-REx cells with stably integrated GFP-SLX4IP were synchronized in either G1, S, G2 or M phase of cell cycle, after which they were harvested for immunoprecipitation of GFP-SLX4IP or for flow cytometry analysis by propidium iodide staining. All synchronizations were performed in parallel, in order to harvest the cells for immunoprecipitation at the same time (which was performed as described above). Doxycycline for the induction of GFP-SLX4IP expression (1 μg/ml final concentration) was added to the cells 24 hours prior to cell harvest. Synchronization for G1 phase sample was obtained by incubation with 40 μM lovastatin for 40 hours. Synchronization for S phase sample was achieved by a double thymidine block and release: cells were first treated with 2 mM thymidine for 17 hours, after which they were washed three times with pre-warmed PBS and were then released from block for 8 hours in fresh media. Thymidine was then added again for another 17 hours, after which the cells were released (as described above) for 3 hours and harvested. Synchronization for G2 phase sample was done by the addition of 9 μM RO-3306 to the media for 20 hours. Synchronization for M phase sample was done by the addition of 50 ng/ml nocodazole to the media for 20 hours, after which the cells were harvested by shake-off. The experiment was performed three times.

#### Biochemical cell fractionation

Biochemical fractionation was performed as previously described in Méndez and Stillman (2000). Briefly, the cells were harvested in PBS using a cell scraper and pelleted at 100 x g for 2 minutes at 4°C. Cells were washed once more in PBS and resuspended in Buffer A (10 mM HEPES-KOH, pH 7.9; 10 mM KCl; 1.5 mM MgCl_2_; 0.34 M sucrose; 10% glycerol; 1 mM DTT; cOmplete, Mini, EDTA-free Protease Inhibitor Cocktail (Sigma-Aldrich)), followed by the addition of Triton X-100 to 0.1% final concentration. The lysate was incubated for 8 minutes on ice before pelleting at 1300 x g for 5 minutes at 4°C. Supernatant (cytoplasmic fraction) was carefully removed from the pellet (nuclei) and clarified by centrifugation at 20000 x g for 5 minutes at 4°C. Nuclei were washed once with Buffer A, before lysis in Buffer B (3 mM EDTA, 0.2 mM EGTA, 1 mM DTT, cOmplete, Mini, EDTA-free Protease Inhibitor Cocktail (Sigma-Aldrich)) for 30 minutes on ice. Insoluble chromatin was pelleted at 1700 x g for 5 minutes at 4°C. Supernatant (nucleoplasm) was carefully separated from the pelleted chromatin, which was then washed once in Buffer B before digestion with Benzonase (Millipore, 2500 U/ml final concentration) in Buffer A (in the cold room on a rotating wheel for 1 hour). Cytoplasmic fraction, nucleoplasm fraction and chromatin digested with benzonase were prepared for SDS-PAGE analysis by the addition of 4x NuPAGE LDS sample buffer (with 89.3 mM 2-mercaptoethanol in final dilution) and boiling for 10 minutes at 95°C.

#### Telomeric chromatin isolation

The pull-down of telomeres was done following a ‘PICh protocol’ (Déjardin and Kingston, 2009, EUROSYS protocol). Briefly, the cells were incubated in a crosslinking solution (1% formaldehyde in 1x PBS) for 30 minutes at room temperature before washing twice in 1x PBS with 1 mM PMSF, scraping cells in 1x PBS with 0.05% Tween-20, and washing again three times in 1x PBS with 1 mM PMSF (washes at 3200 xg and 4°C for 10 minutes). The cell pellets were then frozen at −80°C before continuing with the experiment. Thawed pellets were first washed in a sucrose solution (0.3 M sucrose, 10 mM HEPES-NaOH pH 7.9, 1% Triton X-100, 2 mM MgOAc) and dounced in a 40 mL dounce homogenizer, after which the pellet was washed in a glycerol buffer (25% glycerol, 10 mM HEPES-NaOH pH 7.9, 0.1 mM EDTA, 5 mM MgOAc). Pellets were resuspended in triton solution (0.5% Triton X-100 in 1x PBS) and RNA was digested with the addition of RNase A (1.5 mg/ml; QIAGEN, 19101) overnight at 4°C. Chromatin was then pelleted and washed 6 times in 1x PBS with PMSF, resuspended in high salt lysis buffer (10 mM HEPES-NaOH pH 7.9, 100 mM NaCl, 2 mM EDTA pH 8, 1 mM EGTA pH 8, 0.2% SDS, 0.1% sodium sarkosyl, 1mM PMSF) and sonicated using the Qsonica sonicator Q700 with high power probe. Soluble chromatin was then warmed up to 58°C for 5 minutes and cooled down to room temperature, before adding the samples onto Pierce High Capacity Streptavidin Agarose Resin (equilibrated with high salt lysis buffer; Thermo Fisher Scientific), and incubating them overnight at room temperature on a nutator. Precleared chromatin was added to a dried Sephacryl S-400 HR column (GE Healthcare Life Sciences, 17060901), and then centrifuged once again in 1.5 mL tubes at 16000 x g for 15 minutes at room temperature. After determination of OD260 and OD260/OD280, the precleared and desalted chromatin was supplemented with 0.2% SDS. The samples were then hybridized with 2′F-RNA probes with desthiobiotin (locked nucleic acid, with either scrambled sequence or telomere-specific sequence) in a thermocycler (25°C for 3 minutes, 71°C for 7 minutes, 37°C for 3 hours and then to final temperature of 25°C). Hybridized chromatin was pooled and centrifuged for 15 minutes at 16000 xg at room temperature. Streptavidin magnetic beads Dynabeads MyOne Streptavidine C1 (Thermo Fisher Scientific) were washed twice with low salt lysis buffer (10 mL of buffer per sample; 10 mM HEPES-NaOH pH 7.9, 30 mM NaCl, 2 mM EDTA pH 8, 1 mM EGTA pH 8, 0.2% SDS, 0.1% sodium sarkosyl, 1 mM PMSF), before equal volumes of MilliQ water to chromatin were added to immobilised beads. The chromatin was added to the beads immersed in MilliQ water, followed by an overnight incubation on a nutator at room temperature. Bound chromatin was then washed 6 times with high salt lysis buffer and once with low salt lysis buffer. The beads were resuspended in high salt lysis buffer and transferred into a low protein binding 1.5 mL tube. Immobilised beads were again resuspended in high salt lysis buffer and incubated for 5 minutes at 42°C and 1000 rpm, before repeating it. Washed beads were resuspended in elution buffer (75% high salt lysis buffer, 25% D-biotin; Invitrogen) and the elution was performed at room temperature overnight with shaking at 1000 rpm and for an additional 10 minutes at 65°C without shaking. The eluates were removed from the tubes with immobilised beads and passed twice through new tubes attached to magnetic stand to remove any leftover beads. Eluted proteins were then precipitated with 15%–20% of TCA for 10 minutes at 4°C. Precipitated proteins were pelleted at 16000 xg for 15 minutes at 4°C and the supernatant was removed so that about 200 μL remained above the pellet. Pre-chilled 100% acetone was added to the final volume of 1.5 mL and the sample was then briefly vortexed and pelleted for 10 minutes at 16000 xg and 4°C. The supernatant was fully removed before adding 1.5 mL of cold acetone and repeating the wash. Pellets were air-dried and resuspended in crosslinking reversal solution. Samples were then incubated for 12 minutes at 99°C, before adding Pierce Lane Marker Reducing Sample Buffer (Thermo Fisher Scientific) and incubating for another 13 minutes. Protein samples were then frozen at −80°C until immunoblotting analysis.

#### Recombinant protein production

Recombinant Flag-BLM and MBP-BLM proteins were a kind gift from Andrew Deans (Melbourne, Australia).

cDNA encoding SLX4IP ORF was cloned into Champion pET-SUMO vector (Life Technologies) according to the manufacturer’s instructions. SLX4IP was expressed in *E. coli* BL21(DE3) strain. Protein expression at 18° overnight at OD of 0.7 with 1 mM IPTG. Cells were harvested and resuspended in Lysis buffer (50 mM potassium phosphate (pH 7.8), 1 M KCl, 10% glycerol) supplemented with cOmplete, EDTA-free protease inhibitor cocktail tablets (Roche) (1 tablet per 25 mL buffer), and mixed well with a magnetic stirrer at 4°C until the mixture was homogeneous. The lysate was sonicated on ice using a Branson Sonifier 450. The lysate was then cleared in an Optima LE-80K Ultracentrifuge (Beckman Coulter) using a Ti45 rotor at 20,000 rpm for 60 min at 4°C. Clarified lysate was applied to 5 mL bed volume of Ni-NTA agarose affinity gel (QIAGEN 30210), which had been pre-washed with Lysis Buffer containing 20 mM imidazole. The protein was bound to the beads by rotating at 4°C for 2 h, the flowthrough was discarded and the beads washed with Lysis Buffer containing 20 mM imidazole. The protein was eluted with Lysis Buffer containing 20 mM imidazole containing 100, 200 and 400 mM imidazole and dialyzed against 4 L Dialysis Buffer (20 mM Tris-HCl (pH 8.0), 300 mM KCl, 10% glycerol) overnight using 10 kDa MWCO SnakeSkin dialysis tubing (Thermo Scientific). The His-SUMO tag was cleaved to yield native RAD-51 by addition of 6 μL His-tagged Ulp1 SUMO protease (gift from Peter Cherepanov) for 45 min. Cleaved protein was bound to the same batch of NiNTA agarose affinity gel used for purification after regeneration according to the manufacturer’s instructions to remove the SUMO protease and His-SUMO tag. The flowthrough containing native RASLX4IP was collected and the resin washed with an additional Dialysis Buffer. Remaining protein fraction bound tightly to the beads was eluted by additional imidazole elution step. These were pooled and mixed with Dilution Buffer (20 mM Tris-HCl (pH 8.0), 10% glycerol, 1 mM EDTA, 0.5 mM DTT) to reduce salt concentration to 100 mM KCl. The protein was bound to a 1 mL HiTrap SP column (GE Healthcare) using an Äkta Explorer HPLC system and washed with 10 CV of A buffer (20 mM Tris-HCl (pH 8.0), 10% glycerol, 1 mM EDTA, 0.5 mM DTT, 100 mM KCl). The protein was eluted with a 13 mL 5%–85% Buffer B (20 mM Tris-HCl (pH 8.0), 10% glycerol, 1 mM EDTA, 0.5 mM DTT, 1000 mM KCl) gradient. The peak fractions were pooled, concentrated and frozen in liquid nitrogen.

#### Helicase assay

MBP-BLM in concentrations indicated was incubated in the presence or absence of 100 nM SLX4IP in helicase buffer (25 mM Tris-HCl, pH 7.5; 100 mM NaCl; 5 mM MgOAc; 5 mM ATP; 100 μg/ml BSA; 1 mM DTT and ATP regeneration system consisting of 20 mM creatine phosphate and 20 μg/ml creatine kinase) with 10 nM Y-form substrate (created by annealing oligo y1–[FAM]-AGCTACCATGCCTGCACGAATTAAGCAATTCGTAATCATGGTCAT-AGCT with oligo y2–AGCTATGACCATGATTACGAATTGCTTGGAATCCTGACGAACTGTAG) for 60 minutes at 37°C. Reactions were terminated by addition of 1% SDS and 20 μg of proteinase K and further 15 minute incubation. Reactions were loaded onto 4%–20% gradient PAGE TBE gel and resolved in 1xTBE buffer. Unwinding of synthetic substrate was assessed after scanning the gels on Typhoon9500 instrument.

#### RNA sequencing and gene expression quantification

Total RNA was extracted from the three biological replicates of each cell line using QIAGEN RNeasy Kit according to manufacturer’s instructions for RNA preparation. Samples were submitted to the BU Microarray and Sequencing Core for library preparation and ribosomal RNA reduction using Kapa RNA HyperPrep kit with Riboerase, and sequenced yielding 2 × 75 bp paired-end read datasets.

### Quantification and Statistical Analysis

Statistical analyses (Student’s t test and one-way ANOVA) were performed using PRISM 7 (GraphPad Software). Statistical details of each experiment (including the statistical tests used, exact value of n, what n represents and precision measures) can be found in the figure legends. Brown-Forsythe test was used to determine whether the data met assumptions of the one-way ANOVA analyses.

For RNA sequencing, read library quality was assessed using FastQC and multiqc packages. Illumina adapters were removed and leading and trailing low-quality bases (below quality 30) were trimmed using Trimmomatic. Reads which were less than 36 bases long after these steps were dropped. The expression of the genes was quantified using Salmon with index built from the GENCODE v27 transcriptome. The counts matrix was then normalized using in-house bioinformatics software package de_toolkit’s deseq2 method. The counts of the genes were extracted and plotted with ggplot2 package. The links to all software packages are listed in the Key Resources Table.

### Data and Code Availability

The RNA sequencing data described in this publication have been deposited in NCBI’s Gene Expression Omnibus and are accessible through GEO: GSE124768 (https://www.ncbi.nlm.nih.gov/geo/query/acc.cgi?acc=GSE124768).

## Supplemental Information

Document S1. Figures S1–S7

Document S2. Article plus Supplemental Information
