# Supplementary material for: SLX4IP Antagonizes Promiscuous BLM Activity during ALT Maintenance
Source: Mol Cell. 2019 Oct 3;76(1):27–43.e11. doi: 10.1016/j.molcel.2019.07.010 (PMC6863466; doi:10.1016/j.molcel.2019.07.010)
Supplement: Document S1. Figures S1–S7 [file mmc1.pdf]

Molecular Cell, Volume *76*

Supplemental Information

SLX4IP Antagonizes Promiscuous

BLM Activity during ALT Maintenance

Stephanie Panier, Marija Maric, Graeme Hewitt, Emily Mason-Osann, Himabindu Gali, Anqi Dai, Adam Labadorf, Jean-Hugues Guervilly, Philip Ruis, Sandra Segura-Bayona, Ondrej Belan, Paulina Marzec, Pierre-Henri L. Gaillard, Rachel L. Flynn, and Simon J. Boulton

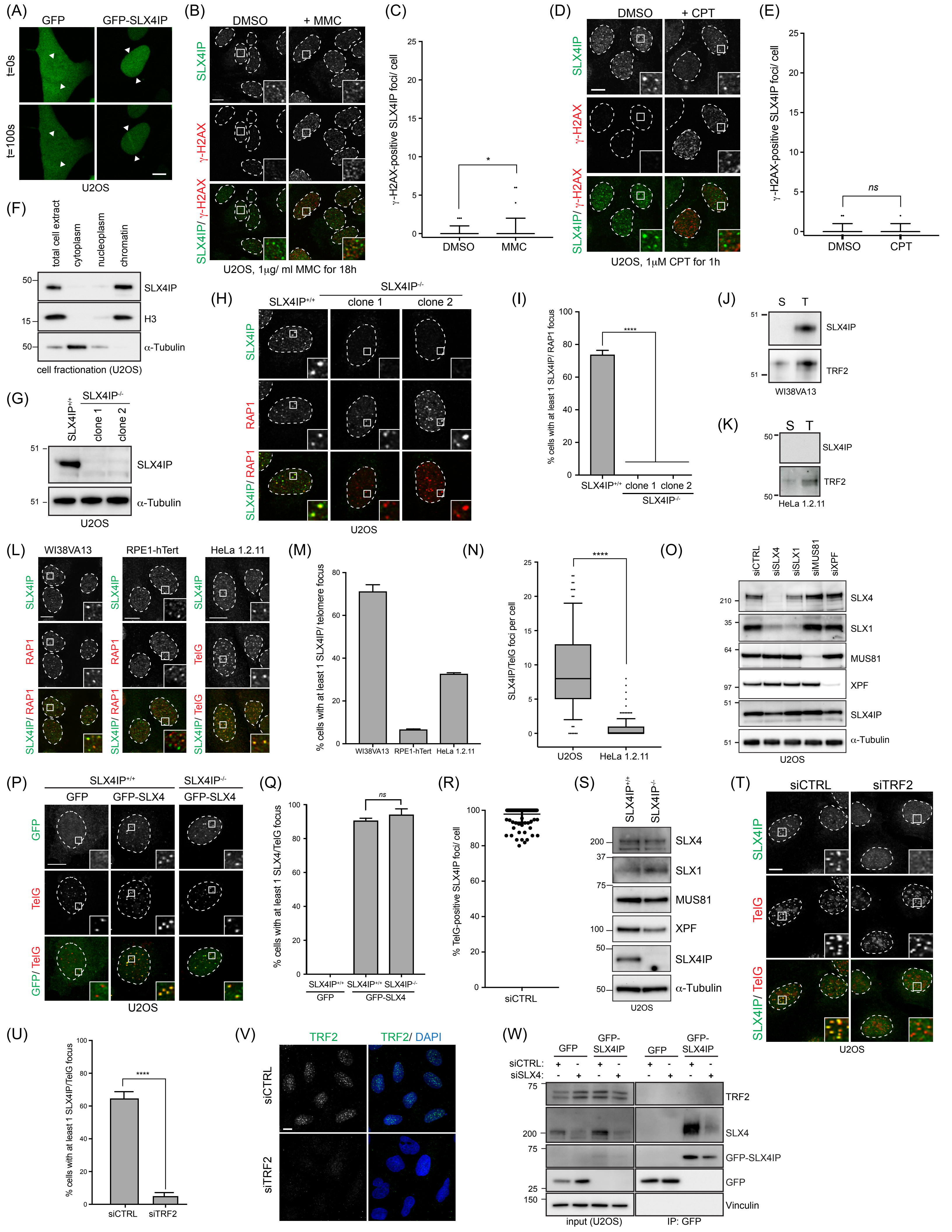
(A)
GFP
GFP-SLX4IP
t=0s
t=100s
U2OS
(B)
DMSO
+ MMC
SLX4IP
γ-H2AX
SLX4IP/ γ-H2AX
U2OS, 1µg/ ml MMC for 18h
(C)
γ-H2AX-positive SLX4IP foci/ cell
*
DMSO
MMC
(D)
DMSO
+ CPT
SLX4IP
γ-H2AX
SLX4IP/ γ-H2AX
U2OS, 1µM CPT for 1h
(E)
γ-H2AX-positive SLX4IP foci/ cell
ns
DMSO
CPT
(F)
total cell extract
cytoplasm
nucleoplasm
chromatin
SLX4IP
H3
α-Tubulin
cell fractionation (U2OS)
(G)
SLX4IP+/+
SLX4IP-/-
clone 1
clone 2
SLX4IP
α-Tubulin
U2OS
(H)
SLX4IP+/+
SLX4IP-/-
clone 1
clone 2
SLX4IP
RAP1
SLX4IP/ RAP1
U2OS
(I)
% cells with at least 1 SLX4IP/ RAP1 focus
****
SLX4IP+/+
clone 1
clone 2
SLX4IP-/-
(J)
S
T
SLX4IP
TRF2
WI38VA13
(K)
S
T
SLX4IP
TRF2
HeLa 1.2.11
(L)
WI38VA13
RPE1-hTert
HeLa 1.2.11
SLX4IP
RAP1
TelG
SLX4IP/ RAP1
SLX4IP/ TelG
(M)
% cells with at least 1 SLX4IP/ telomere focus
WI38VA13
RPE1-hTert
HeLa 1.2.11
(N)
SLX4IP/TelG foci per cell
****
U2OS
HeLa 1.2.11
(O)
siCTRL
siSLX4
siSLX1
siMUS81
siXPF
SLX4
SLX1
MUS81
XPF
SLX4IP
α-Tubulin
U2OS
(P)
SLX4IP+/+
SLX4IP-/-
GFP
GFP-SLX4
GFP-SLX4
GFP
TelG
GFP/ TelG
U2OS
(Q)
% cells with at least 1 SLX4/TelG focus
ns
SLX4IP+/+
SLX4IP+/+
SLX4IP-/-
GFP
GFP-SLX4
(R)
% TelG-positive SLX4IP foci/ cell
siCTRL
(S)
SLX4IP+/+
SLX4IP-/-
SLX4
SLX1
MUS81
XPF
SLX4IP
α-Tubulin
U2OS
(T)
siCTRL
siTRF2
SLX4IP
TelG
SLX4IP/ TelG
U2OS
(U)
% cells with at least 1 SLX4IP/TelG focus
****
siCTRL
siTRF2
(V)
TRF2
TRF2/ DAPI
siCTRL
siTRF2
(W)
GFP
GFP-SLX4IP
GFP
GFP-SLX4IP
siCTRL:
siSLX4:
TRF2
SLX4
GFP-SLX4IP
GFP
Vinculin
input (U2OS)
IP: GFP

Figure S1. Related to Figure 1.

(A) U2OS cells were pre-sensitized with 10μM BrdU and incubated with doxycycline to induce expression of GFP-SLX4IP prior to imaging. Arrows indicate area of laser damage. Scale bar represents 10 μm.

(B) U2OS cells were treated with 1μg/ml mitomycin C (MMC) or DMSO for 18 hours, fixed and processed for SLX4IP and γ-H2AX immunofluorescence. Scale bar represents 10 μm. Dashed lines indicate nucleus outlines (as determined by DAPI staining; not shown). Insets represent 3X magnifications of the indicated fields.

(C) Quantification of (B). At least 100 cells per condition were counted. Data are presented as 5-95 percentile (n=2, Student's t test, * $p<0.01$).

(D) U2OS cells were treated with 1μM camptothecin (CPT) or DMSO for 1 hour, fixed and processed for SLX4IP and γ-H2AX immunofluorescence. Scale bar represents 10 μm. Dashed lines indicate nucleus outlines (as determined by DAPI staining; not shown). Insets represent 3X magnifications of the indicated fields.

(E) Quantification of (D). At least 100 cells per condition were counted. Data are presented as 5-95 percentile (n=2, Student's t test, *ns*, not significant).

(F) Fractionated extracts of U2OS cells separated by SDS-PAGE and analysed by SLX4IP immunoblotting. H3 was used as a chromatin control and α-Tubulin was used as a cytoplasm control. Numbers denote molecular weight (kDa).

(G) U2OS whole-cell extracts were separated by SDS-PAGE and analysed by SLX4IP immunoblotting. Tubulin was used as loading control. Numbers denote molecular weight (kDa).

(H) U2OS cells were fixed and processed for SLX4IP and RAP1 immunofluorescence. Scale bar represents 10 μm. Dashed lines indicate nucleus outlines (as determined by DAPI staining; not shown). Insets represent 3X magnifications of the indicated fields.

(I) Quantification of (H). At least 100 cells per condition were counted. Data are presented as 5-95 percentile (n=3, Student's t test, **** $p<0.00001$).

(J) Chromatin was isolated from whole-cell WI38VA13 extracts with either a scrambled control (S) or a telomere-specific (T) 2'F-RNA probe. The chromatin was separated by SDS-PAGE and analysed by SLX4IP immunoblotting. TRF2 was used as a telomeric chromatin control. Numbers denote molecular weight (kDa).

(K) Chromatin was isolated from whole-cell HeLa 1.2.11 extracts with either a scrambled control (S) or a telomere-specific (T) 2'F-RNA probe. The chromatin was separated by SDS-PAGE and analysed by SLX4IP immunoblotting. TRF2 was used as a telomeric chromatin control. Numbers denote molecular weight (kDa).

(L) WI38VA13 and RPE1 hTERT cells were fixed and processed for SLX4IP and RAP1 immunofluorescence. HeLa 1.2.11 cells were fixed and processed for SLX4IP immunofluorescence followed by telomeric PNA (TelG) FISH. Scale bar represents 10 µm. Dashed lines indicate nucleus outlines (as determined by DAPI staining; not shown). Insets represent 3X magnifications of the indicated fields.

(M) Quantification of (L). At least 100 cells per condition were counted. Data are represented as the mean ± SD (n=3). Note that telomeric localization of SLX4IP in ALT-negative HeLa 1.2.11 cells is significantly lower than in ALT-positive WI38VA13 cells.

(N) Quantification of (Figure S1H, HeLa 1.2.11 and Figure 1A, SLX4IP+/+). At least 100 cells per condition were counted. Data are presented as 5-95 percentile (n=2, Student's t test, **** p<0.00001). Note the number of telomere-positive SLX4IP foci is significantly lower in HeLa 1.2.11 cells compared to U2OS cells, suggesting that the levels of telomere-associated SLX4IP in HeLa 1.2.11 were below the PICh detection limit (see Figure S1K).

(O) Whole-cell extracts of U2OS cells transfected with the indicated siRNAs were separated by SDS-PAGE and analysed by SLX4, SLX1, MUS81, XPF and SLX4IP immunoblotting. α-Tubulin was used as loading control. Numbers denote molecular weight (kDa).

(P) U2OS cells transfected with GFP or GFP-SLX4 were fixed and processed for GFP immunofluorescence followed by telomeric PNA (TelG) FISH. Scale bar represents 10 µm.

Dashed lines indicate nucleus outlines (as determined by DAPI staining; not shown). Insets represent 3X magnifications of the indicated fields.

(Q) Quantification of (P). At least 50 cells per condition were counted. Data are represented as the mean ± SD (n=3, Student's t test, *ns*, not significant).

(R) Quantification of (Figure 1H, siCTRL). At least 100 cells per condition were counted. Data are presented as 5-95 percentile, n=3.

(S) Whole-cell extracts of SLX4IP+/+ and SLX4IP-/- U2OS cells were separated by SDS-PAGE and analysed by SLX4, SLX1, MUS81, XPF and SLX4IP immunoblotting. α-Tubulin was used as loading control. Numbers denote molecular weight (kDa).

(T) U2OS cells transfected with the indicated siRNAs were fixed and processed for SLX4IP immunofluorescence followed by telomeric PNA (TelG) FISH. Scale bar represents 10 µm. Dashed lines indicate nucleus outlines (as determined by DAPI staining; not shown). Insets represent 3X magnifications of the indicated fields.

(U) Quantification of (T). At least 100 cells per condition were counted. Data are represented as the mean ± SD (n=3, Student's t test, **** $p<0.00001$).

(V) Knockdown control of (T). U2OS cells transfected with the indicated siRNAs were fixed and processed for TRF2 immunofluorescence. DNA was counterstained with DAPI. Scale bar represents 10 µm.

(W) Whole-cell extracts from U2OS cells transfected with the indicated siRNAs and transiently expressing the indicated GFP-SLX4IP constructs were subjected to GFP-trap co-immunoprecipitation (IP). Input and IP samples were separated by SDS-PAGE and analysed by GFP, SLX4, and TRF2 immunoblotting. Vinculin was used as loading control. Numbers denote molecular weight (kDa).

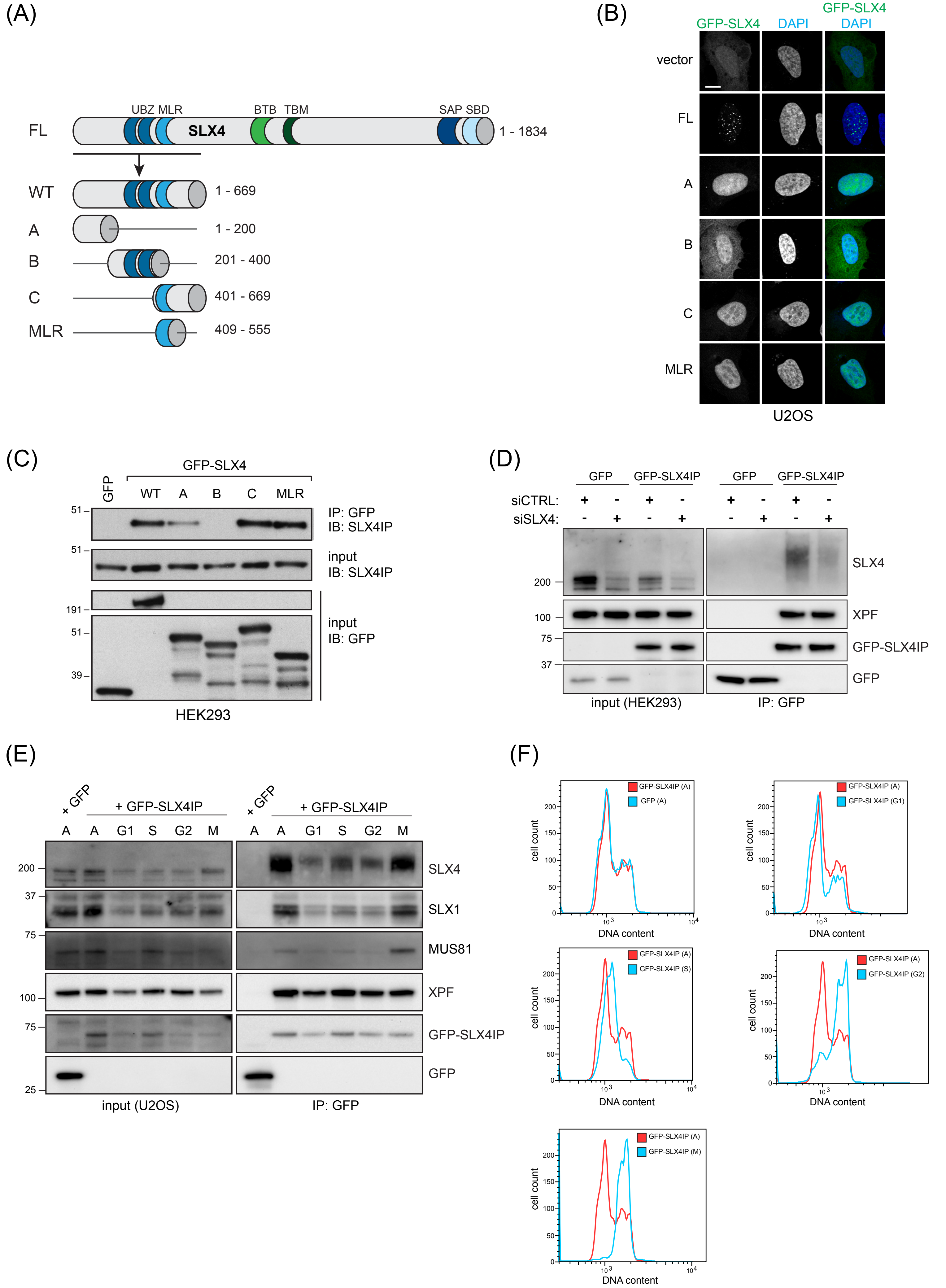

(A)
FL
UBZ MLR
BTB TBM
SAP SBD
SLX4
1 - 1834
WT
1 - 669
A
1 - 200
B
201 - 400
C
401 - 669
MLR
409 - 555
(B)
GFP-SLX4
DAPI
GFP-SLX4 DAPI
vector
FL
A
B
C
MLR
U2OS
(C)
GFP-SLX4
GFP
WT A B C MLR
IP: GFP
IB: SLX4IP
input
IB: SLX4IP
input
IB: GFP
HEK293
(D)
GFP
GFP-SLX4IP
siCTRL:
siSLX4:
SLX4
XPF
GFP-SLX4IP
GFP
input (HEK293)
IP: GFP
(E)
+ GFP
+ GFP-SLX4IP
A A G1 S G2 M
SLX4
SLX1
MUS81
XPF
GFP-SLX4IP
GFP
input (U2OS)
IP: GFP
(F)
GFP-SLX4IP (A)
GFP (A)
GFP-SLX4IP (G1)
GFP-SLX4IP (S)
GFP-SLX4IP (G2)
GFP-SLX4IP (M)
cell count
DNA content

Figure S2. Related to Figure 1.

(A) Domain structures of full-length (FL) SLX4 and of the N-terminal mapping constructs used in (B). Amino acid positions and domains are indicated. WT, wild-type. BTB, broad-complex, tramtrack, and bric à brac; MLR, MUS312-MEI9 interaction-like region; SAP, SAF-A/B, Acinus and PIAS; SBD, SLX1 binding domain; TBM, TRF2-binding motif; UBZ, ubiquitin-binding zinc finger.

(B) Micrographs showing the subcellular localization and expression levels of the constructs depicted in (A). U2OS cells transfected with GFP or the indicated GFP-SLX4 constructs were fixed and processed for GFP immunofluorescence. DNA was counterstained with DAPI. Scale bar represents 10 µm. Domain structures of all constructs are indicated in (A).

(C) Whole-cell extracts from HEK293 cells transiently expressing GFP or the indicated GFP-SLX4 constructs were subjected to GFP-trap co-immunoprecipitation. The co-immunoprecipitates (IP) were separated by SDS-PAGE and analysed by GFP and SLX4IP immunoblotting (IB). Numbers denote molecular weight (kDa). Domain structures of all constructs are indicated in (A).

(D) Whole-cell extracts from HEK293 cells transfected with the indicated siRNAs and transiently expressing GFP or GFP-SLX4IP were subjected to GFP-trap co-immunoprecipitation (IP). Input and IP samples were separated by SDS-PAGE and analysed by GFP, SLX4, and XPF immunoblotting. Numbers denote molecular weight (kDa).

(E) Whole-cell extracts from synchronized U2OS cells inducibly expressing GFP or GFP-SLX4IP were subjected to GFP-trap co-immunoprecipitation (IP). Input and IP samples were separated by SDS-PAGE and analysed by GFP, SLX4, SLX1, MUS81 and XPF immunoblotting. Numbers denote molecular weight (kDa). A, asynchronous; S, S-phase; M, mitosis; G1, G1 phase; G2, G2 phase.

(F) Cell cycle profiles of the synchronized U2OS cells used in (E). Cells were fixed, stained with propidium iodide and analysed by FACS. At least 10 000 cells per condition were counted. The cell cycle stage is indicated in the brackets. A, asynchronous; S, S-phase; M, mitosis; G1, G1 phase; G2, G2 phase.

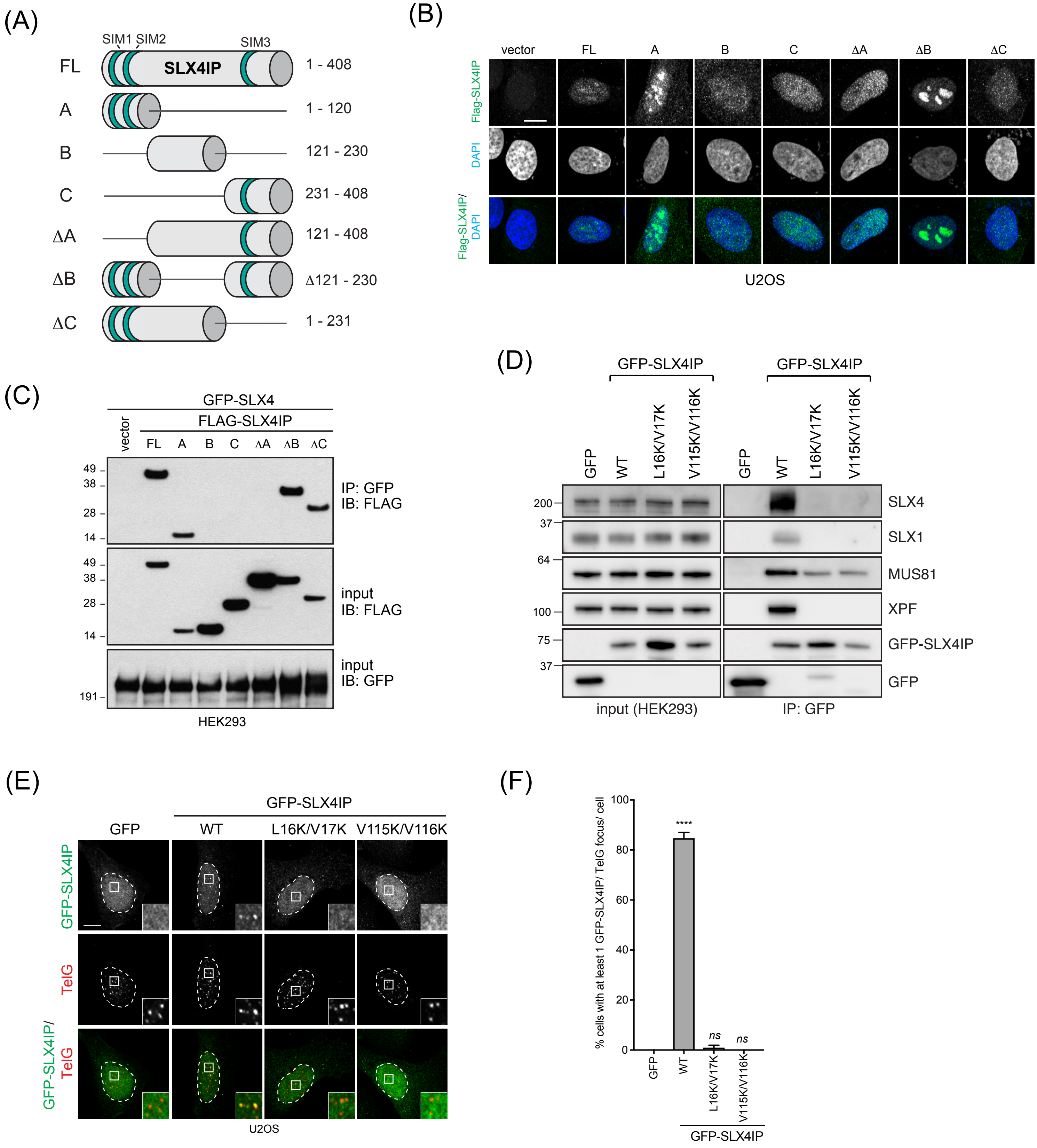
(A)
SIM1 SIM2
SIM3
FL
SLX4IP
1 - 408
A
1 - 120
B
121 - 230
C
231 - 408
ΔA
121 - 408
ΔB
Δ121 - 230
ΔC
1 - 231
(B)
vector FL A B C ΔA ΔB ΔC
Flag-SLX4IP
DAPI
Flag-SLX4IP/ DAPI
U2OS
(C)
GFP-SLX4
FLAG-SLX4IP
vector FL A B C ΔA ΔB ΔC
49
38
28
14
IP: GFP
IB: FLAG
input
IB: FLAG
input
IB: GFP
191
HEK293
(D)
GFP-SLX4IP
GFP-SLX4IP
GFP WT L16K/V17K V115K/V116K GFP WT L16K/V17K V115K/V116K
200
37
64
100
75
37
SLX4
SLX1
MUS81
XPF
GFP-SLX4IP
GFP
input (HEK293)
IP: GFP
(E)
GFP-SLX4IP
GFP WT L16K/V17K V115K/V116K
GFP-SLX4IP
TelG
GFP-SLX4IP/ TelG
U2OS
(F)
% cells with at least 1 GFP-SLX4IP/ TelG focus/ cell
100
80
60
40
20
0
****
ns
ns
GFP
WT
L16K/V17K
V115K/V116K
GFP-SLX4IP

Figure S3. Related to Figure 1.

(A) Domain structures of full-length (FL) SLX4IP and of the mapping constructs used in (B). Amino acid positions and domains are indicated. SIM, SUMO interacting motif.

(B) Micrographs showing the subcellular localization and expression levels of the constructs depicted in (A). U2OS cells transfected with a vector control or the indicated Flag-SLX4IP constructs were fixed and processed for Flag immunofluorescence. DNA was counterstained with DAPI. Scale bar represents 10 μm. Domain structures of all constructs are indicated in (A).

(C) Whole-cell extracts from HEK293 cells stably expressing GFP-SLX4 and transiently expressing the indicated FLAG-SLX4IP constructs were subjected to GFP-trap co-immunoprecipitation. The co-immunoprecipitates (IP) were separated by SDS-PAGE and analysed by GFP and FLAG immunoblotting (IB). Numbers denote molecular weight (kDa). Domain structures of all constructs are indicated in (A).

(D) Whole-cell extracts from HEK293 cells transfected with GFP or the indicated GFP-SLX4IP constructs were subjected to GFP-trap co-immunoprecipitation (IP). Input and IP samples were separated by SDS-PAGE and analysed by GFP, SLX4, SLX1, MUS81 and XPF immunoblotting. Numbers denote molecular weight (kDa). WT, wild type.

(E) U2OS cells transiently transfected with the indicated GFP-SLX4IP constructs were fixed and processed for GFP immunofluorescence followed by telomeric PNA (TelG) FISH. Scale bar

represents 10 μm. Dashed lines indicate nucleus outlines (as determined by DAPI staining; not shown). Insets represent 3 X magnifications of the indicated fields. WT, wild type.

(F) Quantification of (E). At least 100 cells per condition were counted. Data are represented as the mean ± SD (n=3, Student's t test, **** $p<0.00001$, *ns*, not significant).

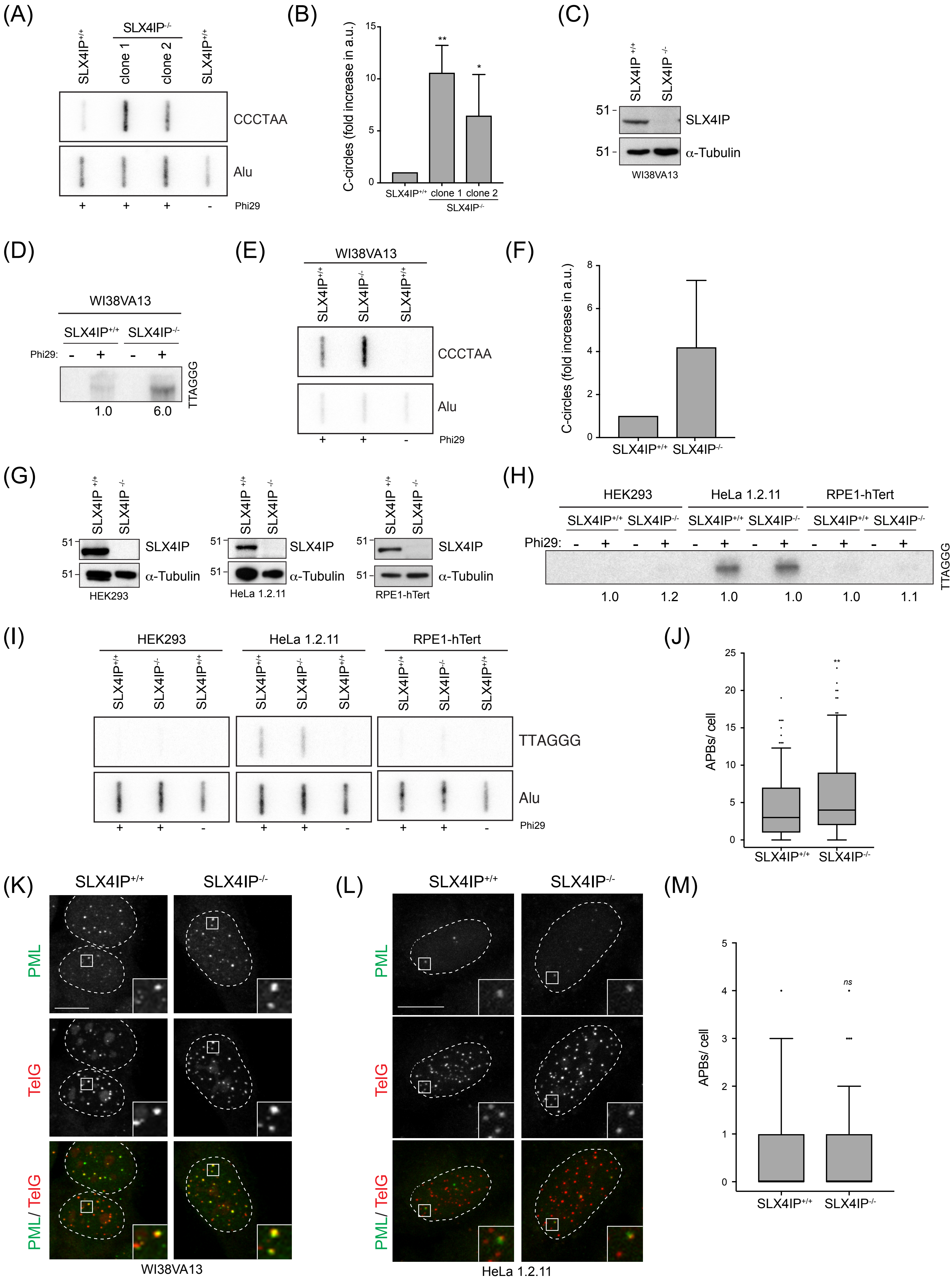

(A)
SLX4IP+/+
SLX4IP-/-
clone 1
clone 2
SLX4IP+/+
CCCTAA
Alu
Phi29
(B)
C-circles (fold increase in a.u.)
**
*
SLX4IP+/+
clone 1
clone 2
SLX4IP-/-
(C)
SLX4IP +/+
SLX4IP -/-
51
SLX4IP
α-Tubulin
WI38VA13
(D)
WI38VA13
SLX4IP+/+
SLX4IP-/-
Phi29:
TTAGGG
1.0
6.0
(E)
WI38VA13
SLX4IP+/+
SLX4IP-/-
SLX4IP+/+
CCCTAA
Alu
Phi29
(F)
C-circles (fold increase in a.u.)
SLX4IP+/+
SLX4IP-/-
(G)
SLX4IP +/+
SLX4IP -/-
SLX4IP
α-Tubulin
HEK293
HeLa 1.2.11
RPE1-hTert
(H)
HEK293
HeLa 1.2.11
RPE1-hTert
SLX4IP+/+
SLX4IP-/-
Phi29:
TTAGGG
1.0
1.2
1.0
1.0
1.0
1.1
(I)
HEK293
HeLa 1.2.11
RPE1-hTert
SLX4IP+/+
SLX4IP-/-
TTAGGG
Alu
Phi29
(J)
APBs/ cell
**
SLX4IP+/+
SLX4IP-/-
(K)
SLX4IP+/+
SLX4IP-/-
PML
TelG
PML/ TelG
WI38VA13
(L)
SLX4IP+/+
SLX4IP-/-
PML
TelG
PML/ TelG
HeLa 1.2.11
(M)
APBs/ cell
ns
SLX4IP+/+
SLX4IP-/-

Figure S4. Related to Figure 2.

(A) Genomic DNA was isolated from U2OS cells and processed to detect Phi29-dependent C-circles. The Phi29 amplification products were detected by Southern blotting using a $\gamma[^{32}P]$-labelled telomeric (CCCTAA) probe. An Alu probe was used as loading control.

(B) Quantification of (A). The extent of $[^{32}P]$-incorporation was quantified from the autoradiograph and normalized to SLX4IP+/+, which was arbitrarily assigned a value of 1. Data are represented as the mean ± SD (n=3, Student's t test, * $p<0.01$, ** $p<0.001$). a.u., arbitrary units.

(C) Whole-cell extracts of WI38VA13 cells were separated by SDS-PAGE and analysed by SLX4IP immunoblotting. α-Tubulin was used as loading control. Numbers denote molecular weight (kDa).

(D) Genomic DNA was isolated from WI38VA13 cells and processed to detect Phi29-dependent telomere circles. The Phi29 amplification products were detected by Southern blotting using a $\gamma[^{32}P]$-labelled telomeric (TTAGGG) probe. Numbers indicate fold increase of $[^{32}P]$-incorporation relative SLX4IP+/+, which was arbitrarily assigned a value of 1.

(E) Genomic DNA was isolated from WI38VA13 cells and processed to detect Phi29-dependent C-circles. The Phi29 amplification products were detected by Southern blotting using a $\gamma[^{32}P]$-labelled telomeric (CCCTAA) probe. An Alu probe was used as loading control.

(F) Quantification of (E). The extent of [^{32}P]-incorporation was quantified from the autoradiograph and normalized to SLX4IP+/+, which was arbitrarily assigned a value of 1. Data are represented as the mean ± SD (n=2). a.u., arbitrary units.

(G) Whole-cell extracts of RPE1 hTERT, HeLa 1.2.11 and HEK293 cells were separated by SDS-PAGE and analysed by SLX4IP immunoblotting. α-Tubulin was used as loading control. Numbers denote molecular weight (kDa).

(H) Genomic DNA was isolated from RPE1 h-TERT, HeLa 1.2.11 and HEK293 cells and processed to detect Phi29-dependent telomere circles. The Phi29 amplification products were detected by Southern blotting using a γ[^{32}P]-labelled telomeric (TTAGGG) probe. Numbers indicate fold increase of [^{32}P]-incorporation relative SLX4IP+/+, which was arbitrarily assigned a value of 1.

(I) Genomic DNA was isolated from RPE1 h-TERT, HeLa 1.2.11 and HEK293 cells and processed to detect Phi29-dependent C-circles. The Phi29 amplification products were detected by Southern blotting using a γ[^{32}P]-labelled telomeric (CCCTAA) probe. An Alu probe was used as loading control.

(J) WI38VA13 cells were fixed and processed for PML immunofluorescence followed by telomeric PNA (TelG) FISH. At least 100 cells per condition were counted. Data are presented as 5-95 percentile (n=3, Student's t test, ** $p<0.001$).

(K) Micrograph images of (J). Scale bar represents 10 μm. Dashed lines indicate nucleus outlines (as determined by DAPI staining; not shown). Insets represent 3X magnifications of the indicated fields.

(L) HeLa 1.2.11 cells were fixed and processed for PML immunofluorescence followed by telomeric PNA (TelG) FISH. Scale bar represents 10 μm. Dashed lines indicate nucleus outlines (as determined by DAPI staining; not shown). Insets represent 3X magnifications of the indicated fields.

(M) Quantification of (L). At least 100 cells per condition were counted. Data are presented as 5-95 percentile (n=3, Student's t test; *ns*, not significant).

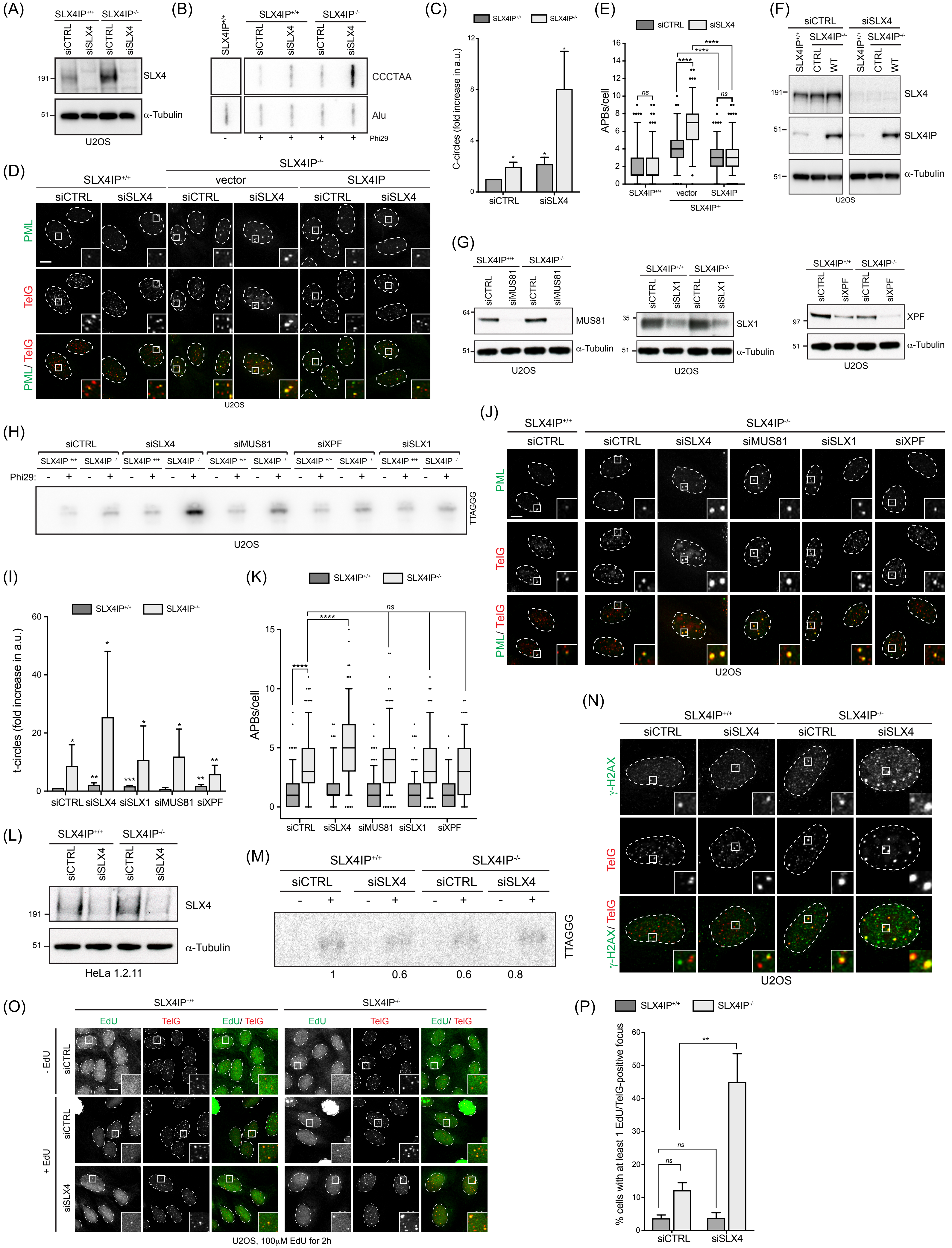

(A)
SLX4IP+/+
SLX4IP-/-
siCTRL
siSLX4
191
SLX4
51
α-Tubulin
U2OS
(B)
SLX4IP+/+
CCCTAA
Alu
Phi29
(C)
C-circles (fold increase in a.u.)
siCTRL
siSLX4
(D)
vector
SLX4IP
PML
TelG
PML/ TelG
U2OS
(E)
APBs/cell
ns
****
vector
(F)
CTRL
WT
SLX4IP
(G)
siMUS81
MUS81
siSLX1
SLX1
siXPF
XPF
(H)
Phi29:
TTAGGG
(I)
t-circles (fold increase in a.u.)
(J)
(K)
(L)
HeLa 1.2.11
(M)
1
0.6
0.6
0.8
(N)
γ-H2AX
γ-H2AX/ TelG
(O)
EdU
EdU/ TelG
- EdU
+ EdU
U2OS, 100μM EdU for 2h
(P)
% cells with at least 1 EdU/TelG-positive focus

Figure S5. Related to Figure 3.

(A) Whole-cell extracts of U2OS cells transfected with the indicated siRNAs were separated by SDS-PAGE and analysed by SLX4. α-Tubulin was used as loading control. Numbers denote molecular weight (kDa).

(B) Genomic DNA was isolated from U2OS cells transfected siRNAs and processed to detect Phi29-dependent C-circles. The Phi29 amplification products were detected by Southern blotting using a $\gamma[^{32}P]$-labelled telomeric (CCCTAA) probe. An Alu probe was used as loading control.

(C) Quantification of (B). The extent of $[^{32}P]$-incorporation was quantified from the autoradiograph and normalized to SLX4IP+/+ siCTRL, which was arbitrarily assigned a value of 1. Data are represented as the mean ± SD (n=3, Student's t test, * $p<0.01$, ** $p<0.001$). a.u., arbitrary units.

(D) U2OS cells transfected with the indicated siRNAs were fixed and processed for PML immunofluorescence followed by telomeric PNA (TelG) FISH. Scale bar represents 10 μm. Dashed lines indicate nucleus outlines (as determined by DAPI staining; not shown). Insets represent 3X magnifications of the indicated fields.

(E) Quantification of (D). At least 100 cells per condition were counted. Data are presented as 5-95 percentile (n=3, one-way ANOVA, **** $p<0.00001$; *ns*, not significant).

(F) Whole-cell extracts of U2OS cells transfected with the indicated siRNAs were separated by SDS-PAGE and analysed by SLX4IP immunoblotting. α-Tubulin was used as loading control. Numbers denote molecular weight (kDa). Note that lanes irrelevant for the figure were removed. This is indicated by a space between the siCTRL and siSLX4 conditions.

(G) Whole-cell extracts of U2OS cells transfected with the indicated siRNAs were separated by SDS-PAGE and analysed by SLX1, MUS81 and XPF immunoblotting. α-Tubulin was used as loading control. Numbers denote molecular weight (kDa).

(H) Genomic DNA was isolated from U2OS cells transfected with the indicated siRNAs and processed to detect Phi29-dependent telomere circles. The Phi29 amplification products were detected by Southern blotting using a $\gamma[^{32}P]$-labelled telomeric (TTAGGG) probe.

(I) Quantification of (H). The extent of $[^{32}P]$-incorporation was quantified from the autoradiograph and normalized to SLX4IP+/+ siCTRL, which was arbitrarily assigned a value of 1. Data are represented as the mean ± SD (n=3, Student's t test, * $p<0.01$). a.u., arbitrary units.

(J) U2OS cells transfected with the indicated siRNAs were fixed and processed for PML immunofluorescence followed by telomeric PNA (TelG) FISH. Scale bar represents 10 µm. Dashed lines indicate nucleus outlines (as determined by DAPI staining; not shown). Insets represent 3X magnifications of the indicated fields.

(K) Quantification of (J). At least 100 cells per condition were counted. Data are presented as 5-95 percentile (n=3, one-way ANOVA, **** $p<0.00001$; *ns*, not significant).

(L) Whole-cell extracts of HeLa 1.2.11 cells transfected with the indicated siRNAs were separated by SDS-PAGE and analysed by SLX4 immunoblotting. Tubulin was used as loading control. Numbers denote molecular weight (kDa).

(M) Genomic DNA was isolated from HeLa 1.2.11 cells transfected with the indicated siRNAs and processed to detect Phi29-dependent telomere circles. The Phi29 amplification products were detected by Southern blotting using a $\gamma[^{32}P]$-labelled telomeric (TTAGGG) probe. Numbers indicate fold increase of $[^{32}P]$-incorporation relative SLX4IP+/+ siCTRL, which was arbitrarily assigned a value of 1.

(N) U2OS cells transfected with the indicated siRNAs were fixed and processed for γ-H2AX immunofluorescence followed by telomeric PNA (TelG) FISH. Scale bar represents 10 µm. Dashed lines indicate nucleus outlines (as determined by DAPI staining; not shown). Insets represent 3X magnifications of the indicated fields.

(O) U2OS cells transfected with the indicated siRNAs were treated with 100 µM 5-ethynyl-2′-deoxyuridine (EdU) for 2 hours, fixed and processed for EdU immunofluorescence followed by telomeric PNA (TelG) FISH. Scale bar represents 10 µm. Dashed lines indicate nucleus outlines (as determined by DAPI staining; not shown). Insets represent 3X magnifications of the indicated fields.

(P) Quantification of (O). At least 100 cells per condition were counted. Data are presented as 5-95 percentile (n=3, one-way ANOVA, ** p<0.001, *ns*, not significant).

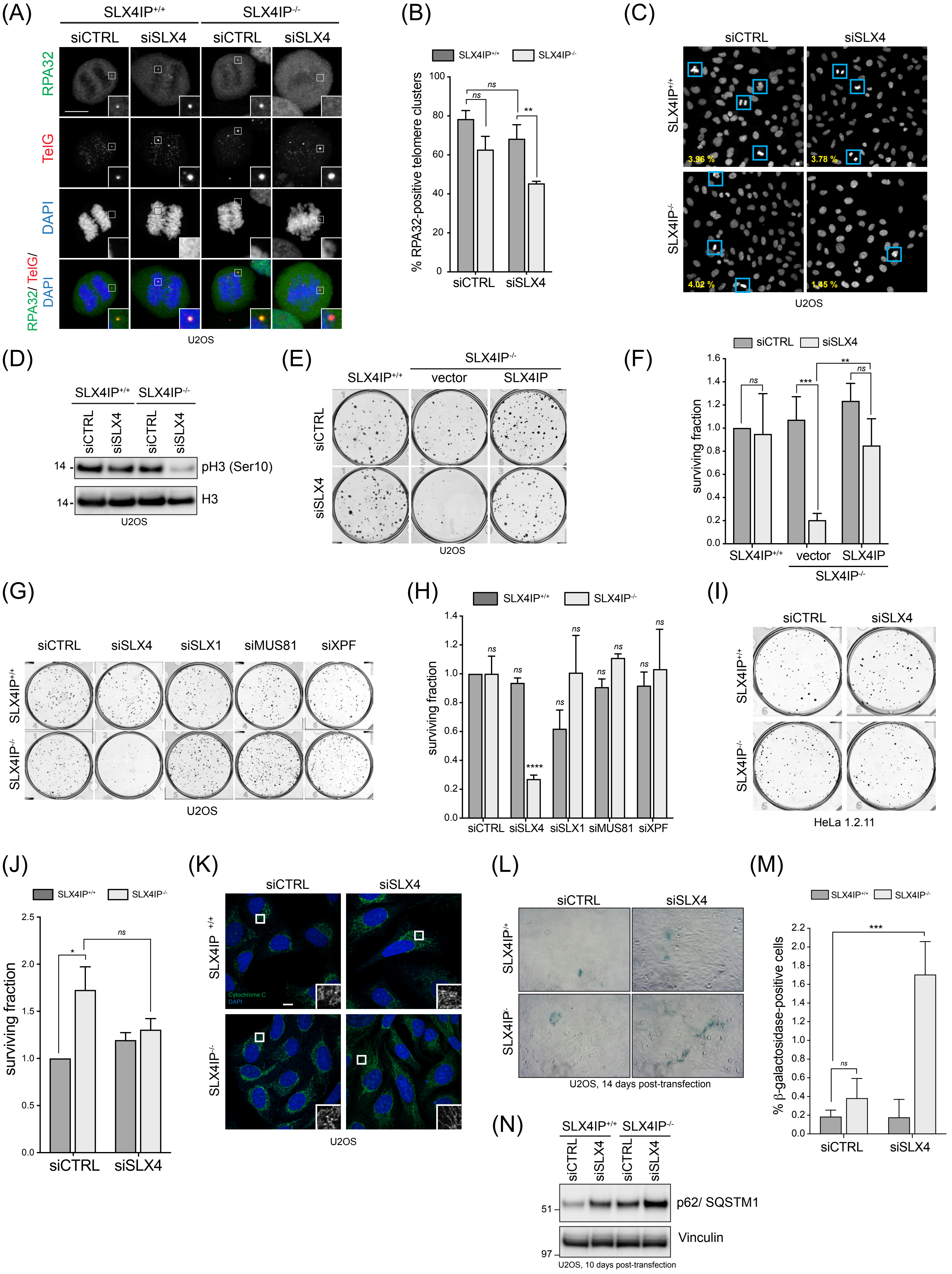
(A)
SLX4IP+/+
SLX4IP-/-
siCTRL
siSLX4
siCTRL
siSLX4
RPA32
TelG
DAPI
RPA32/ TelG/ DAPI
U2OS
(B)
SLX4IP+/+
SLX4IP-/-
% RPA32-positive telomere clusters
100
80
60
40
20
0
ns
ns
**
siCTRL
siSLX4
(C)
siCTRL
siSLX4
SLX4IP+/+
SLX4IP-/-
3.96 %
3.78 %
4.02 %
1.45 %
U2OS
(D)
SLX4IP+/+
SLX4IP-/-
siCTRL
siSLX4
siCTRL
siSLX4
14
pH3 (Ser10)
14
H3
U2OS
(E)
SLX4IP-/-
SLX4IP+/+
vector
SLX4IP
siCTRL
siSLX4
U2OS
(F)
siCTRL
siSLX4
surviving fraction
1.6
1.4
1.2
1.0
0.8
0.6
0.4
0.2
0.0
ns
***
**
ns
SLX4IP+/+
vector
SLX4IP
SLX4IP-/-
(G)
siCTRL
siSLX4
siSLX1
siMUS81
siXPF
SLX4IP+/+
SLX4IP-/-
U2OS
(H)
SLX4IP+/+
SLX4IP-/-
surviving fraction
1.4
1.2
1.0
0.8
0.6
0.4
0.2
0.0
ns
****
siCTRL
siSLX4
siSLX1
siMUS81
siXPF
(I)
siCTRL
siSLX4
SLX4IP+/+
SLX4IP-/-
HeLa 1.2.11
(J)
SLX4IP+/+
SLX4IP-/-
surviving fraction
2.5
2.0
1.5
1.0
0.5
0.0
*
ns
siCTRL
siSLX4
(K)
siCTRL
siSLX4
SLX4IP +/+
SLX4IP-/-
Cytochrome C
DAPI
U2OS
(L)
siCTRL
siSLX4
SLX4IP+/+
SLX4IP-/-
U2OS, 14 days post-transfection
(M)
SLX4IP+/+
SLX4IP-/-
% β-galactosidase-positive cells
2.2
2.0
1.8
1.6
1.4
1.2
1.0
0.8
0.6
0.4
0.2
0.0
***
ns
siCTRL
siSLX4
(N)
SLX4IP+/+
SLX4IP-/-
siCTRL
siSLX4
siCTRL
siSLX4
51
p62/ SQSTM1
97
Vinculin
U2OS, 10 days post-transfection

Figure S6. Related to Figure 4.

(A) U2OS cells transfected with the indicated siRNAs were fixed and processed for RPA32 immunofluorescence followed by telomeric PNA (TelG) FISH. DNA was counterstained with DAPI. Scale bar represents 10 μm. Insets represent 3X magnifications of the indicated fields.

(B) Quantification of (A). At least 100 cells per condition were counted. Data are presented as 5-95 percentile (n=3, one-way ANOVA, ** $p<0.001$, *ns*, not significant).

(C) U2OS cells transfected with the indicated siRNAs were fixed and processed for DAPI staining. Scale bar represents 10 μm. Blue boxes indicate mitotic cells. Numbers indicate mitotic index.

(D) Whole-cell extracts of U2OS cells transfected with the indicated siRNAs were separated by SDS-PAGE and analysed by pH3 (Ser10) immunoblotting. α-Tubulin was used as loading control. Numbers denote molecular weight (kDa).

(E) U2OS cells were transfected with the indicated siRNAs. After 72 hours of knockdown, cells were re-seeded and were then permitted to grow for 11 days before fixation and staining.

(F) Quantification of (E). The surviving fraction was normalized to SLX4IP+/+, which was arbitrarily assigned a value of 1. Data are represented as the mean ± SD (n=3, Student's t test, *** $p<0.0001$, ** $p<0.001$, *ns*, not significant).

(G) U2OS cells were transfected with the indicated siRNAs. After 72 hours of knockdown, cells were re-seeded and were then permitted to grow for 11 days before fixation and staining.

(H) Quantification of (G). The surviving fraction was normalized to SLX4IP+/+ siCTRL, which was arbitrarily assigned a value of 1. Data are represented as the mean ± SD (n=3, Student's t test, *** $p<0.0001$, ** $p<0.001$, *ns*, not significant).

(I) HeLa 1.2.11 cells were transfected with the indicated siRNAs. After 72 hours of knockdown, cells were re-seeded and were then permitted to grow for 9 days before fixation and staining.

(J) Quantification of (I). The surviving fraction was normalized to SLX4IP+/+ siCTRL, which was arbitrarily assigned a value of 1. Data are represented as the mean ± SD (n=3, Student's t test, * $p<0.01$, *ns*, not significant).

(K) U2OS cells transfected with the indicated siRNAs were fixed and processed for Cytochrome C immunofluorescence. DNA was counterstained with DAPI. Scale bar represents 10 µm. Insets represent 3X magnifications of the indicated fields.

(L) U2OS cells were transfected with the indicated siRNAs. After 72 hours of knockdown, cells were re-seeded and were then permitted to grow for 11 days before fixation and β-galactosidase staining.

(M) Quantification of (L). At least 1500 cells per condition were counted. Data are presented as 5-95 percentile (n=3, one-way ANOVA, *** $p<0.0001$; *ns*, not significant).

(N) Whole-cell extracts of U2OS cells transfected with the indicated siRNAs were separated by SDS-PAGE and analysed by p62/ SQSTM1 immunoblotting. Vinculin was used as loading control. Numbers denote molecular weight (kDa).

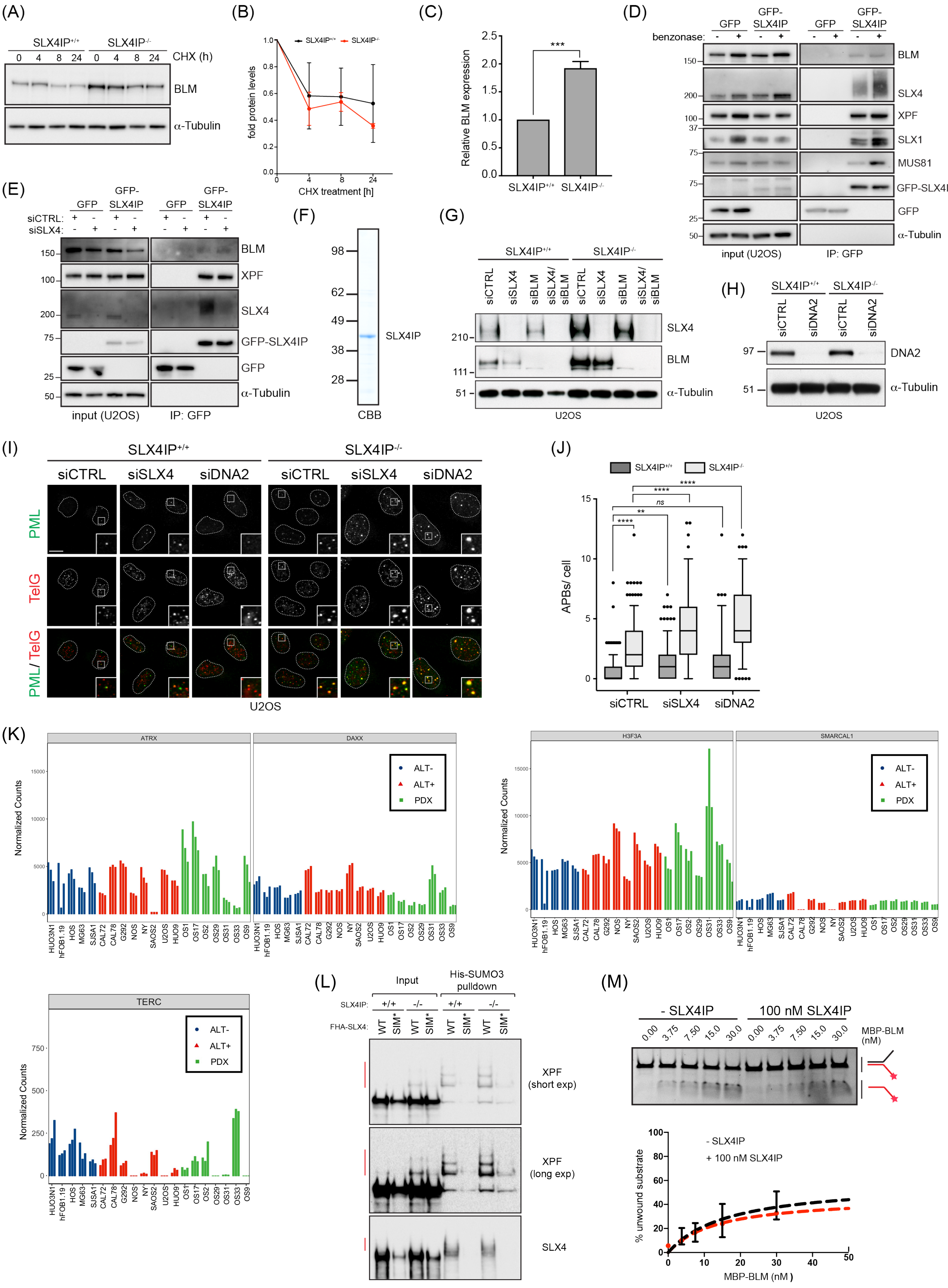

(A)
SLX4IP+/+
SLX4IP-/-
0 4 8 24 0 4 8 24
CHX (h)
BLM
α-Tubulin
(B)
SLX4IP+/+
SLX4IP-/-
fold protein levels
CHX treatment [h]
(C)
Relative BLM expression
***
SLX4IP+/+
SLX4IP-/-
(D)
GFP
GFP-SLX4IP
benzonase:
BLM
SLX4
XPF
SLX1
MUS81
GFP-SLX4IP
GFP
α-Tubulin
input (U2OS)
IP: GFP
(E)
GFP
GFP-SLX4IP
siCTRL:
siSLX4:
BLM
XPF
SLX4
GFP-SLX4IP
GFP
α-Tubulin
input (U2OS)
IP: GFP
(F)
SLX4IP
CBB
(G)
SLX4IP+/+
SLX4IP-/-
siCTRL
siSLX4
siBLM
siSLX4/siBLM
SLX4
BLM
α-Tubulin
U2OS
(H)
SLX4IP+/+
SLX4IP-/-
siCTRL
siDNA2
DNA2
α-Tubulin
U2OS
(I)
SLX4IP+/+
SLX4IP-/-
siCTRL
siSLX4
siDNA2
PML
TelG
PML/ TelG
U2OS
(J)
SLX4IP+/+
SLX4IP-/-
APBs/ cell
****
**
ns
siCTRL
siSLX4
siDNA2
(K)
ATRX
DAXX
H3F3A
SMARCAL1
Normalized Counts
ALT-
ALT+
PDX
TERC
(L)
Input
His-SUMO3 pulldown
SLX4IP:
FHA-SLX4:
WT
SIM*
XPF (short exp)
XPF (long exp)
SLX4
(M)
- SLX4IP
100 nM SLX4IP
MBP-BLM (nM)
% unwound substrate
+ 100 nM SLX4IP
MBP-BLM (nM)

Figure S7. Related to Figure 4, Figure 6 and Figure 7.

(A) Whole-cell extracts of U2OS cells treated with 20 µg/ ml cycloheximide (CHX) for the indicated time points were separated by SDS-PAGE and analysed by BLM immunoblotting. α-Tubulin was used as loading control. Numbers denote molecular weight (kDa).

(B) Quantification of (A). Data were normalized to t=0, which were arbitrarily assigned a value of 1. Data are represented as the mean ± SD, n=3.

(C) *BLM* gene expression in the indicated cell lines was analysed by RT-qPCR. Data were normalized to SLX4IP+/+, which was arbitrarily assigned a value of 1. Data are represented as the mean ± SD (n=3, Student's t test, *** $p<0.0001$).

(D) Benzonase-treated whole-cell extracts from HEK293 cells transfected with GFP or GFP-SLX4IP were subjected to GFP-trap co-immunoprecipitation (IP). Input and IP samples were separated by SDS-PAGE and analysed by GFP, BLM and SLX4, SLX1, MUS81 and XPF immunoblotting. α-Tubulin was used as loading control. Numbers denote molecular weight (kDa).

(E) Whole-cell extracts from U2OS cells transiently transfected with the indicated siRNAs and GFP or GFP-SLX4IP were subjected to GFP-trap co-immunoprecipitation (IP). Input and IP samples were separated by SDS-PAGE and analysed by GFP, BLM, SLX4 and XPF immunoblotting. α-Tubulin was used as loading control. Numbers denote molecular weight (kDa).

(F) Recombinant SLX4IP was purified and analysed by SDS-PAGE and Coomassie Brilliant Blue (CBB) staining. Numbers denote molecular weight (kDa).

(G) Whole-cell extracts of U2OS cells transfected with the indicated siRNAs were separated by SDS-PAGE and analysed by SLX4 and BLM immunoblotting. Tubulin was used as loading control. Numbers denote molecular weight (kDa).

(H) Whole-cell extracts of U2OS cells transfected with the indicated siRNAs were separated by SDS-PAGE and analysed by DNA2 immunoblotting. Tubulin was used as loading control. Numbers denote molecular weight (kDa).

(I) U2OS cells transfected with the indicated siRNAs were fixed and processed for PML immunofluorescence followed by telomeric PNA (TelG) FISH. Scale bar represents 10 μm. Dashed lines indicate nucleus outlines (as determined by DAPI staining; not shown). Insets represent 3X magnifications of the indicated fields.

(J) Quantification of (I). At least 100 cells per condition were counted. Data are presented as 5-95 percentile (n=2, one-way ANOVA, **** $p<0.0001$, ** $p<0.001$, *ns*, not significant).

(K) *ATRX, DAXX, SMARCAL1, H3.3* and *Terc* gene expression in osteosarcoma cell lines and tumours.

(L) U2OS cells transiently transfected with the indicated FHA-SLX4 constructs and with His-SUMO3 were subjected to denaturing His-pulldowns. The pulldowns were separated by SDS-PAGE and analysed by XPF and SLX4 immunoblotting. Red lines indicate SUMOylated XPF and SLX4.

(M) Recombinant MBP-BLM was incubated in the presence or absence of 100 nM SLX4IP in helicase buffer with 10 nM Y-form substrate. Reactions were resolved in a 4-20% gradient PAGE TBE gel.
